# Synthesis, photophysical, computational, and cytotoxicity evaluation of carborane-appended phenylene and triazine trimers: elucidating the role of the central core

**DOI:** 10.1039/d6ra04231g

**Published:** 2026-06-01

**Authors:** Simran Pattnaik, Anwesha Pradhan, Laxmipriya Nayak, Subhadeep Acharya, Supriya Routray, Subhasri Dan, Soumya Ranjan Jena, Kshatresh Dutta Dubey, Luna Samanta, Rashmirekha Satapathy

**Affiliations:** a Department of Chemistry, Ravenshaw University Cuttack-753003 Odisha India rashmi@ravenshawuniversity.ac.in; b Department of Zoology, Ravenshaw University Cuttack-753003 Odisha India; c Department of Chemistry, School of Natural Sciences, Shiv Nadar Institution of Eminence Delhi NCR India

## Abstract

The development of tumor-selective boron carriers is critical for advancing targeted cancer therapies. In this study, we report the design, synthesis, and characterization of four *ortho*-carborane-appended symmetrical trimers, Ph-6-CB, Ph-9-CB, Tz-6-CB, and Tz-9-CB, to systematically compare the influence of central-core electronics and peripheral carborane density on photophysical and biological properties. Photophysical studies revealed that all four conjugates exhibit strong π–π* absorption in the 328–335 nm region; the triazine-cored derivatives show a modest blue shift relative to their phenylene analogs. Phenylene derivatives retain higher fluorescence quantum yields than their triazine analogs. Qualitative DFT analysis displays smaller HOMO–LUMO separations and substantially higher computed electrophilicity indices for triazine analogs than the phenylene analogs. Preliminary *in vitro* cytotoxicity assays against MDA-MB-231 triple-negative breast cancer cells and NIH/3T3 mouse embryo fibroblasts revealed higher potency and greater cancer selectivity of triazine derivatives than their phenylene counterparts. Tz-9-CB emerged as the lead candidate, with an IC_50_ of 6 µM against MDA-MB-231 cells and a selectivity index of 13 relative to NIH/3T3 fibroblasts. Mechanistic studies, including Live/Dead fluorescence imaging and caspase-3 activation assays, confirmed that Tz-9-CB induces cell death primarily through apoptosis. These findings highlight the potential of triazine-cored, carborane-rich dendrimers as selective scaffolds for boron-based cancer therapeutics.

## Abstract 2

Four *ortho*-carborane symmetrical trimers were synthesized and evaluated. The triazine-cored derivative Tz-9-CB selectively kills MDA-MB-231 TNBC cells (IC_50_ = 6 µM, SI = 13 *vs* NIH/3T3) by inducing caspase-3-mediated apoptosis.

## Graphical abstract

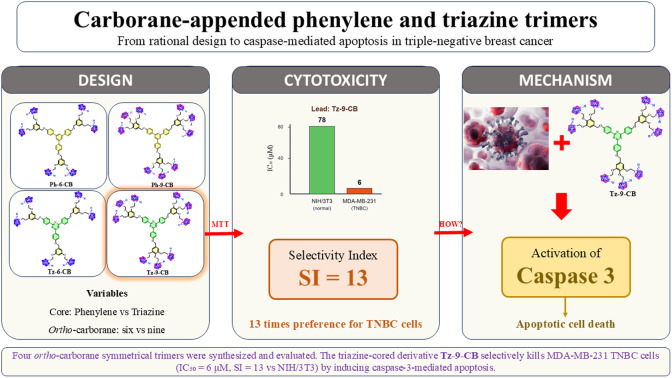

## 1. Introduction

**Fig. 1:**
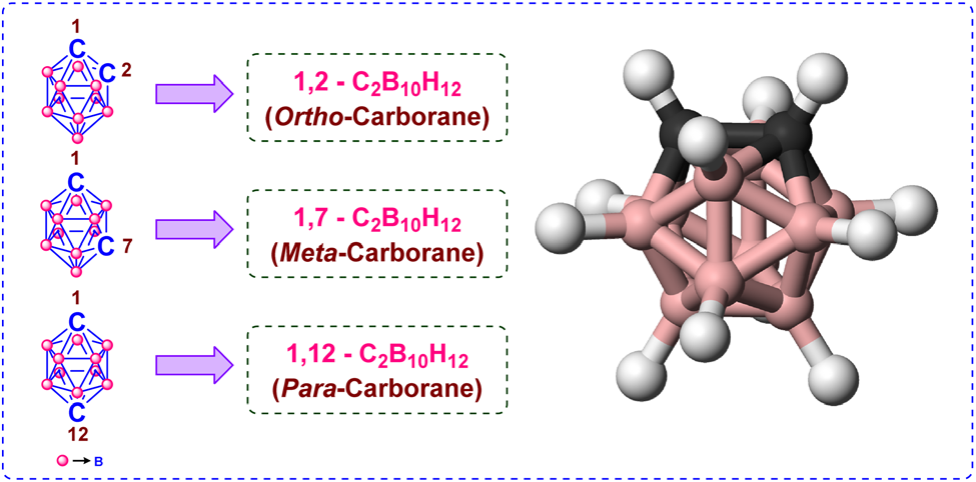
Structure of *ortho*-, *meta*-, and *para*-carborane.

Icosahedral carboranes constitute a distinctive family of polyhedral boron-carbon clusters that are regarded as three-dimensional analogs of benzene because of their delocalized sigma-electron bonding.^1–4^ Among these, dicarba-*closo*-dodecaborane (*closo*-carborane) is the most common structural variant within the carborane family. This compound exists in three structural isomers, distinguished by the relative positions of the two carbon atoms within the icosahedral framework (Fig. 1). The rigid *closo*-carborane cage exhibits remarkable chemical and thermal stability, pronounced resistance toward hydrolysis, and inherent hydrophobicity.^5^ Owing to these attributes, carboranes have been extensively explored across diverse research domains, including medicinal chemistry^6–17^ (*e.g.*, drug design and boron neutron capture therapy), materials science^18–24^ (*e.g.*, polymer engineering and nanomaterials), and coordination chemistry^25–31^ (*e.g.*, ligand design and catalysis).

The architectural design of the molecular scaffold plays a pivotal role in determining the efficacy of carborane-based conjugates. Symmetrical star-shaped or dendritic molecules offer the advantage of multivalency, where the spatial arrangement of multiple bioactive units on a central core can lead to synergistic enhancements in binding affinity and biological activity.^32^ Dendrimers can exploit the enhanced permeability and retention (EPR) effect, enabling preferential accumulation in tumor tissues.^33^ In this context, the selection of the central core is critical. The 1,3,5-triazine ring is a privileged scaffold in drug discovery, known for its electron-deficient nature and its ability to participate in hydrogen bonding, often serving as a core for various anticancer and antimicrobial agents.^34^ In contrast, phenylene cores provide a widely used, electronically neutral, and chemically robust platform for comparison.^35^ Elucidating the role of the central core's electronic nature, specifically electron-deficient triazine compared to neutral phenylene, in governing the photophysical and biological behavior of carborane-functionalized dendrimers continues to attract considerable research attention.

Breast cancer, a leading cause of cancer-related deaths worldwide, is marked by the uncontrolled growth of abnormal cells in the breast.^36,37^ Triple-negative breast cancer (TNBC) is a type of breast cancer identified by the absence of estrogen receptors (ER), progesterone receptors (PR), and the lack of HER-2 amplification.^38^ Globally, TNBC makes up about 10–24% of all breast cancer cases and is more common in Asian countries.^39^ Despite advances in prevention, early detection, and treatment, the worldwide incidence of breast cancer continues to increase.^40^

In this study, we report the design, synthesis, and characterization of four *ortho*-carborane appended symmetrical trimers containing phenylene and triazine cores. We synthesized two series of compounds: phenylene-cored derivatives (Ph-6-CB, Ph-9-CB) and triazine-cored derivatives (Tz-6-CB, Tz-9-CB), bearing six and nine peripheral *ortho*-carborane cages, respectively. While dendritic architectures bearing peripheral *ortho*-carborane cages have been reported previously,^41–43^ a systematic comparison of how the electronic character of the central aromatic core interacts with peripheral carborane density to govern photophysical and biological behavior has not been undertaken. We employed Density Functional Theory (DFT) and Time-Dependent DFT (TD-DFT) to probe the frontier molecular orbitals. The photophysical properties, including the extinction coefficients (*ε*_max_) and quantum yield, of the trimers were examined using absorption and fluorescence spectroscopy in spectroscopic-grade acetone. Furthermore, the *in vitro* cytotoxicity of these conjugates was evaluated against the human breast adenocarcinoma cell line MDA-MB-231 and the mouse embryo fibroblast cell line NIH/3T3. Remarkably, the triazine-based dendrimers demonstrated superior potency and exceptional selectivity compared to cisplatin. Finally, we investigated the mechanism of cell death, confirming that these conjugates induce apoptosis *via* Live/Dead fluorescence imaging and the activation of the caspase-3 pathway.

## 2 Results and discussion

### 2.1 Synthesis

#### 2.1.1 Synthesis of carborane dendrons 5a and 5b

**Scheme 1:**
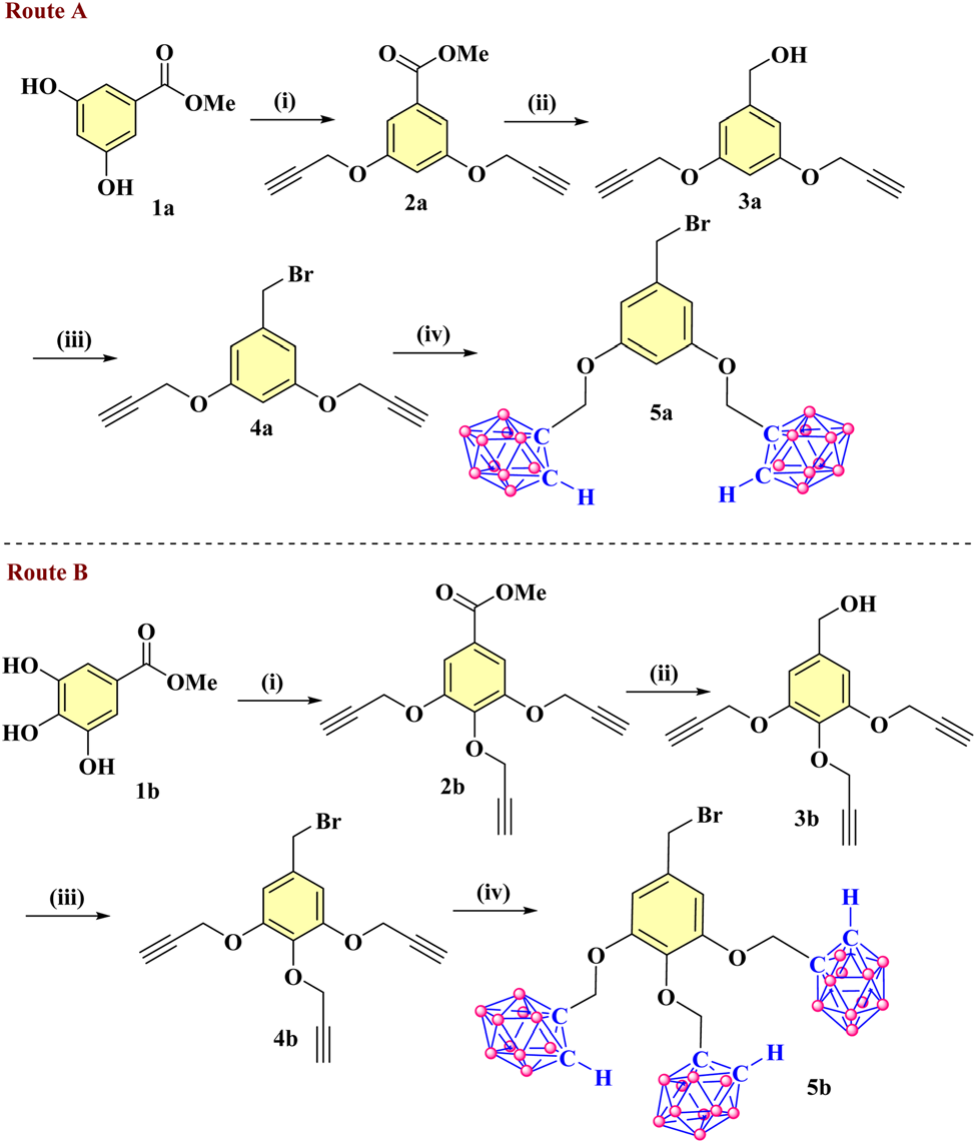
Synthesis of the di-carboranyl dendron 5a (Route A) and the tri-carboranyl dendron 5b (Route B). (i) propargyl bromide, K_2_CO_3_, dry acetone, reflux at 80 °C, 20 h (yields: 1a 89%; 1b 86%); (ii) LiAlH_4_, dry THF, rt, 7 h (yields: 3a 88%; 3b 81%); (iii) PBr_3_, dry CH_2_Cl_2_, rt, 15 h (yields: 4a 85%; 4b 82%); (iv) decaborane(B_10_H_14_), dry CH_3_CN, reflux at 90 °C, 12 h, (yields: 5a 31%; 5b 31%).

**Scheme 2:**
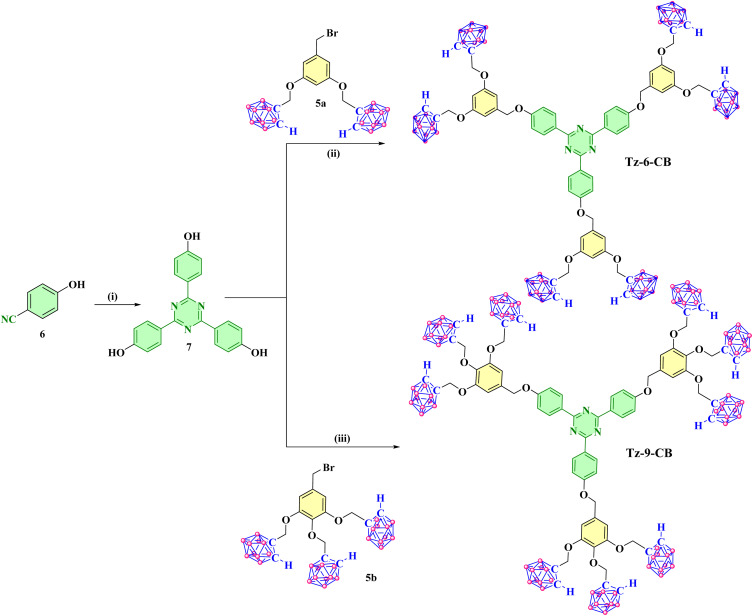
Synthesis of the *ortho*-carborane-appended triazine-cored trimers Tz-6-CB and Tz-9-CB. (i) CF_3_SO_3_H, dry CHCl_3_, rt, 24 h, 87%; (ii) K_2_CO_3_, dry acetone, reflux at 60 °C, 16 h, 44%; (iii) K_2_CO_3_, dry acetone, reflux at 60 °C, 36 h, 33%.

**Scheme 3:**
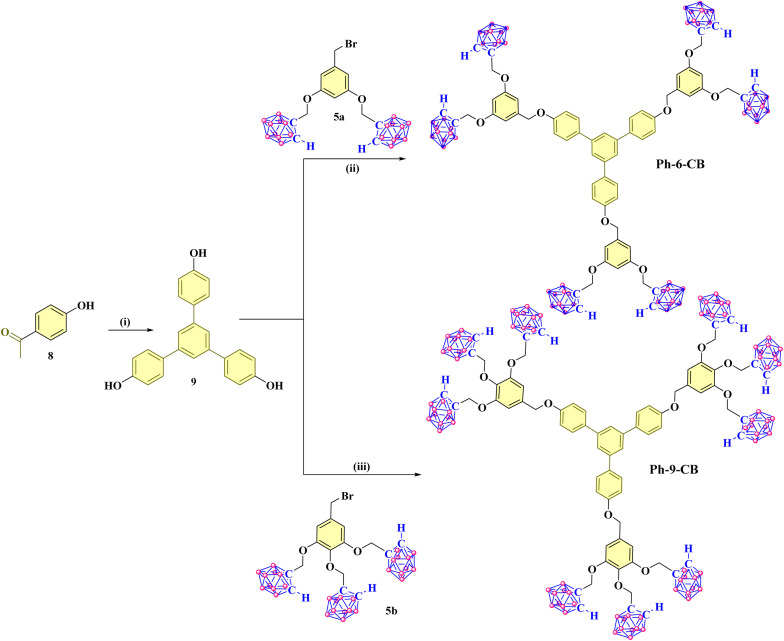
Synthesis of the *ortho*-carborane-appended phenylene-cored trimers Ph-6-CB and Ph-9-CB.(i) SiCl_4_, dry EtOH/Toluene, rt, 71%; (ii) K_2_CO_3_, dry acetone, reflux at 60 °C, 16 h, 42%; (iii) K_2_CO_3_, dry acetone, reflux at 60 °C, 36 h, 36%.

Four carborane-appended symmetrical trimers containing phenylene and triazine cores were successfully synthesized. First, the alkyne dendrons 4a and 4b were synthesized from commercially available methyl-3,5-dihydroxybenzoate and methyl-3,4,5-trihydroxybenzoate, following the literature procedure.^7^ Compounds 4a and 4b react with decaborane in acetonitrile to give *ortho*-carborane dendrons 5a and 5b, respectively, as a white solid with a 31% yield (Scheme 1). Further, the triazine-cored derivatives (Tz-6-CB, Tz-9-CB) and phenylene-cored derivatives (Ph-6-CB, Ph-9-CB) bearing six and nine peripheral *ortho*-carborane cages, respectively, were synthesized following a series of reactions (Scheme 2 and 3).

#### 2.1.2 Synthesis of *ortho*-carborane appended trimers having triazine core (Compound Tz-6-CB and Tz-9-CB)

The compounds Tz-6-CB and Tz-9-CB were synthesized through a series of reactions starting from the commercially available 4-hydroxybenzonitrile 6. The starting material compound 6 undergoes cyclotrimerization in the presence of triflic acid, producing the triazine-cored derivative 2,4,6-Tris(*p*-hydroxyphenyl) triazine 7 with a yield of 87% (Scheme 2).^42^ Compound 7 undergoes condensation reaction with *ortho*-carborane dendrons 5a and 5b in the presence of K_2_CO_3_ to produce *ortho*-carborane appended trimers having triazine core Tz-6-CB and Tz-9-CB, respectively. Both trimers, Tz-6-CB and Tz-9-CB, were characterized by IR, NMR, and MALDI-TOF mass spectrometric analyses (see SI). The IR spectra exhibited characteristic B–H stretching frequencies at 2565 cm^−1^ and 2591 cm^−1^ for the compounds Tz-6-CB and Tz-9-CB, respectively. The ^1^H NMR spectra displayed broad singlet signals at *δ* 5.33 ppm for compound Tz-6-CB (Fig. S7) and at *δ* 5.24 and 5.08 ppm for compound Tz-9-CB (Fig. S10), confirming the presence of the *ortho*-carborane cage in both symmetrical trimers. Finally, the MALDI-TOF spectral analysis showed a peak at *m*/*z* 1661.2662 [M]^+^ for the compound Tz-6-CB (Fig. S21) and *m*/*z* 2194.4403 [M]^+^ for the compound Tz-9-CB (Fig. S22), confirming their formation.

#### 2.1.3 Synthesis of *ortho*-carborane appended trimers having phenylene core (Compound Ph-6-CB and Ph-9-CB)

The compounds Ph-6-CB and Ph-9-CB, having a phenylene core were synthesized through a series of reactions starting from the commercially available 4-hydroxyacetophenone 8. The starting material, compound 8, undergoes a SiCl4–mediated cyclotrimerization, producing a phenylene-cored derivative, 9, as a colorless powder in 71% yield (Scheme 3). Compounds Ph-6-CB and Ph-9-CB were synthesized by the condensation reaction with *ortho*-carborane dendrons 5a and 5b in the presence of K_2_CO_3_, respectively. Both phenylene-cored trimers, Ph-6-CB and Ph-9-CB, were characterized by IR, NMR, and mass spectrometric analyses (see SI). The IR spectra exhibited a characteristic B–H stretching frequency at 2583 cm^−1^ and 2588 cm^−1^ for the compounds Ph-6-CB and Ph-9-CB, respectively. The ^1^H NMR displayed broad singlet signals at *δ* 4.05 ppm for compound Ph-6-CB (Fig. S13) and *δ* 4.09 ppm, 3.96 ppm for compound Ph-9-CB (Fig. S16), which confirms the presence of the *ortho*-carborane cage in both the symmetrical trimers. Finally, the MALDI-TOF spectral analysis showed a peak at *m*/*z* 1658.4625 [M]^+^ for the compound Ph-6-CB (Fig. S23) and *m*/*z* 2174.7013 [M]^+^ for the compound Ph-9-CB (Fig. S24), confirming their formation.

### 2.2 Photophysical studies

**Fig. 2:**
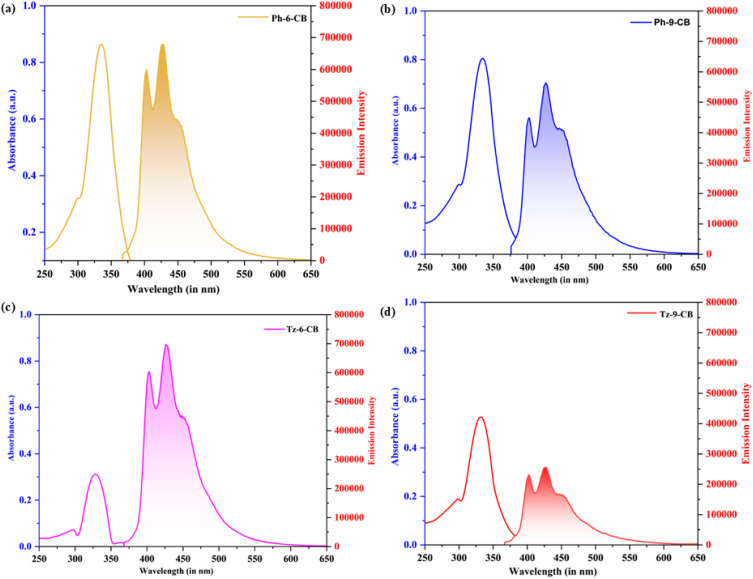
Absorption and normalized fluorescence spectra of (a) Ph-6-CB, (b) Ph-9-CB, (c) Tz-6-CB and (d) Tz-9-CB in 1.0 × 10^−5^ M solution of acetone.

The photophysical properties of the phenylene- and triazine-cored dendrimers bearing six and nine *ortho*-carborane cages were investigated in pure spectroscopic-grade acetone at an analyte concentration of 1.0 × 10^−5^ M (Fig. 2). Our objective was to elucidate the influence of the triazine and phenylene cores, as well as peripheral carborane incorporation, on the optical behaviour of the molecule. All four dendrimers display intense absorption bands in the 328–335 nm region characteristic of π–π* transitions localized on the central aromatic core (Table 1 and Fig. 2). The phenylene-cored derivatives absorb at slightly longer wavelengths (335 and 334 nm for Ph-6-CB and Ph-9-CB, respectively) than their triazine-cored analogues (332 and 328 nm for Tz-6-CB and Tz-9-CB). The modest blue shift of the main π–π* absorption band observed for the triazine series arises from the intrinsic electrophilicity of the 1,3,5-triazine core, whose three ring nitrogens significantly lower the π-electron density and stabilize the ground state more strongly than the π–π* excited state. This differential stabilization of the ground state relative to the excited state increases the vertical excitation energy and produces the small hypsochromic shift observed experimentally. Within each series, increasing the number of peripheral *ortho*-carborane cages from six to nine induces a further minor blue shift (1 nm for the phenylene series, 4 nm for the triazine series), consistent with a small steric perturbation of cross-conjugation between the central aromatic core and the outer dendron arms.

**Table 1:** UV-vis absorption and steady-state fluorescence emission data of Ph-6-CB, Ph-9-CB, Tz-6-CB, and Tz-9-CB. All measurements were performed in spectroscopic-grade acetone at a concentration of 1.0 × 10^−5^ M and at 25 °C. *λ*_abs_ = absorption maximum; *λ*_em_ = fluorescence emission maxima; *λ*_ex_ = 360 nm; *λ*_C_ = crossover wavelength between absorption and emission bands; relative area = integrated area beneath the fluorescence emission band; *Φ*_F_ = relative fluorescence quantum yield; *ε* = molar extinction coefficient

Substrates	Solvent	Absorption maxima (nm)	Emission (nm)	Relative area	Quantum yield (*ϕ*_F_)	*λ*_C_ (nm)	Stokes shift (nm)	Extinct coeff, *ε* (10^4^ M^−1^ cm^−1^)
Ph-6-CB	Acetone	335	403, 427	0.54	0.30	375	92	8.66
Ph-9-CB	Acetone	334	402, 427	0.45	0.25	383	93	8.05
Tz-6-CB	Acetone	332	403, 426	0.54	0.30	368	94	3.12
Tz-9-CB	Acetone	328	402, 427	0.21	0.12	382	99	5.27

All dendrimers exhibit dual emission features centered at 402 nm and 426 nm, which remain largely invariant across the series. To determine relative fluorescence quantum yields (*Φ*_F_), the dendrimers were excited at 360 nm. To overcome the challenges associated with measuring absolute quantum yields, it is common to assess the quantum yields of unknown samples by comparison with a standard of known fluorescence quantum yield.^44^ The quantum yield of compound Quinine Sulfate is reported to be 0.55.^45^ Using these values, the relative fluorescence quantum yields (*Φ*_F_'s) of phenylene and triazine dendrimers are calculated by analyzing the area beneath the fluorescence bands (Table 1). The phenylene-cored dendrimers retain relatively strong emission, with Ph-6-CB showing the highest fluorescence output (*Φ*_F_ = 0.30, relative area 0.54). A moderate decrease in *Φ*_F_ is observed for Ph-9-CB (*Φ*_F_ = 0.25), attributable to increased steric hindrance from the additional *ortho*-carborane cages that facilitates nonradiative relaxation. A more pronounced effect is observed for the triazine-based dendrimers. While Tz-6-CB exhibits a quantum yield identical to its phenylene analogue (*Φ*_F_ = 0.30), the nine *ortho*-carborane derivative Tz-9-CB undergoes substantial fluorescence quenching (*Φ*_F_ = 0.12). This trend is further supported by variations in the extinction coefficient, where phenylene-based dendrimers Ph-6-CB (*ε* = 8.66 × 10^4^ M^−1^ cm^−1^) and Ph-9-CB (*ε* = 8.05 × 10^4^ M^−1^ cm^−1^) show stronger absorption than their triazine counterparts Tz-6-CB (*ε* = 3.12 × 10^4^ M^−1^ cm^−1^) and Tz-9-CB (*ε* = 5.27 × 10^4^ M^−1^ cm^−1^), highlighting the reduced conjugation efficiency in the triazine series.

All four dendrimers display large Stokes shifts in the range of 92–99 nm, indicating significant excited-state geometric relaxation. The highest Stokes shift is observed for Tz-9-CB (99 nm), in agreement with its enhanced excited-state reorganization and lowest emission efficiency. The *λ*_c_ values (368–383 nm) follow similar trends, reflecting subtle core-dependent variations. Collectively, these results demonstrate that the central core plays a dominant role in governing the photophysical behavior of these dendrimers. Phenylene-cored systems maintain higher radiative efficiency, whereas the triazine-cored dendrimers, especially Tz-9-CB, undergo substantial fluorescence quenching due to higher steric crowding and non-radiative decay.

### 2.3 Density functional theory (DFT) calculations

To complement the experimental photophysical and biological characterization of the four trimers, density functional theory (DFT) and time-dependent DFT (TD-DFT) calculations were performed on Ph-6-CB, Ph-9-CB, Tz-6-CB, and Tz-9-CB. The calculations outlined in this section are intended as a qualitative illustration of electronic-structure trends throughout the series, specifically, the spatial localization of frontier molecular orbitals, the relative ordering of HOMO–LUMO separations between the phenylene- and triazine-cored systems, the dominant character of the lowest singlet excitations, and the qualitative electrostatic potential distribution. We do not attempt quantitative comparison between computed and experimentally observed absorption maxima, nor do we draw mechanistic conclusions from absolute numerical values.

We note that the B3LYP functional combined with a 6-31G basis set is an approximate model for boron-rich dendritic systems and for excitations with charge-transfer character, and that modern carborane computational studies typically employ polarized triple-zeta basis sets (*e.g.*, 6-311G*) and long-range-corrected functionals for quantitatively predictive treatment.^46^ A quantitatively predictive treatment of these systems would require, at minimum, conformational sampling of the multiple rotamers accessible to the dendritic framework at finite temperature, polarized and diffuse basis sets, a long-range-corrected exchange-correlation functional, and relaxation of the lowest excited-state geometries. Such treatment is beyond the scope of the present study, and the calculations are accordingly used here only for qualitative trends and descriptors.

**Fig. 3:**
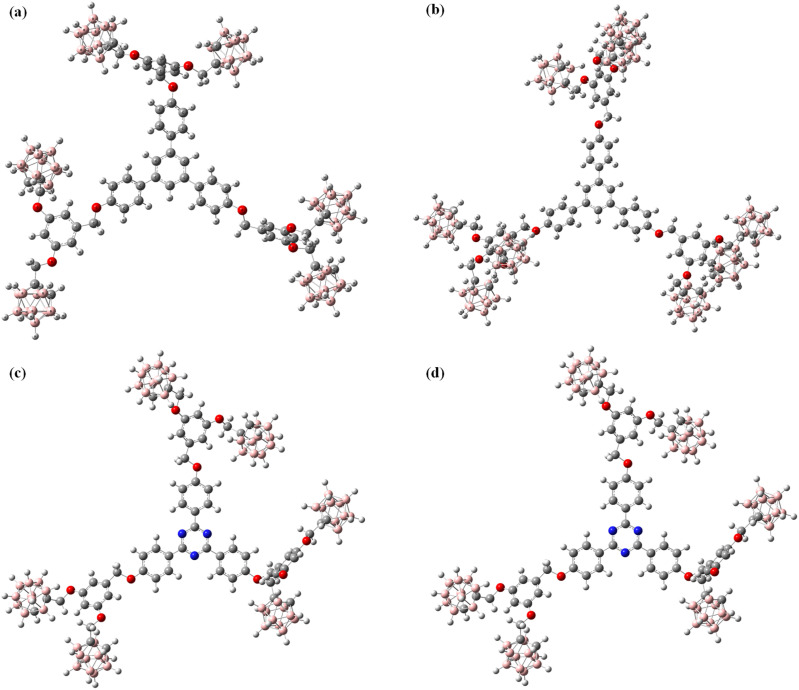
Optimized geometries of (a) Ph-6-CB, (b) Ph-9-CB, (c) Tz-6-CB and (d) Tz-9-CB at B3LYP/6-31G level. The structures shown represent local minima obtained without conformational sampling.

Ground-state geometry optimizations were performed at the B3LYP/6-31G level without symmetry constraints, followed by harmonic vibrational frequency analyses confirming that all optimized geometries correspond to true minima on the potential energy surface (no imaginary frequencies). Representative optimized geometries are shown in Fig. 3.

#### 2.3.1 Frontier molecular orbital analysis

Frontier molecular orbital (FMO) analysis was carried out to examine the spatial distribution of the HOMO and LUMO across the series, and to evaluate qualitative trends in HOMO–LUMO energy separation and derived conceptual DFT descriptors.^47^ We emphasize that the absolute orbital energies and gap values reported in this section are model-dependent; they should be interpreted at the level of relative trends across the series rather than as quantitatively converged values.

**Fig. 4:**
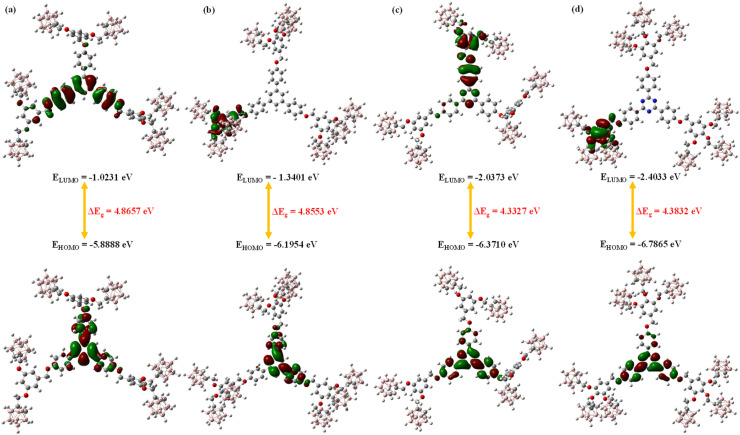
Qualitative HOMO and LUMO surface maps of (a) Ph-6-CB, (b) Ph-9-CB, (c) Tz-6-CB, and (d) Tz-9-CB at the B3LYP/6-31G level.

For all four compounds, the HOMO is predominantly localized over the central π-conjugated aromatic framework, while the LUMO is distributed across the peripheral *ortho*-carborane cages. This pattern, which is consistent across the four compounds, reflects the electron-donating character of the aromatic core and the σ-aromatic, electron-accepting character of the icosahedral *ortho*-carborane cluster. The qualitative HOMO and LUMO surfaces are shown in Fig. 4, and the corresponding orbital energies at the B3LYP/6-31G level are summarized in Table 2.

**Table 2:** Frontier molecular orbital energies (in eV) of Ph-6-CB, Ph-9-CB, Tz-6-CB, and Tz-9-CB computed at the B3LYP/6-31G level. Absolute orbital energies are model-dependent and are presented to illustrate relative trends across the series

Substrate	Computed energies (in eV) of the frontier molecular orbitals							
	H	H − 1	H − 2	H − 3	L	L + 1	L + 2	L + 3
Ph-6-CB	−5.8888	−5.9440	−6.3059	−6.4575	−1.0231	−0.9855	−0.9110	−0.8351
Ph-9-CB	−6.1954	−6.2499	−6.6188	−7.0455	−1.3401	−1.3129	−1.2860	−1.2536
Tz-6-CB	−6.3710	−6.4398	−6.4673	−6.6190	−2.0373	−2.0163	−0.9771	−0.8767
Tz-9-CB	−6.7865	−6.8061	−6.8382	−7.1174	−2.4033	−2.3845	−1.3700	−1.3515

The phenylene-cored compounds show larger HOMO–LUMO separations (computed Δ*E*_g_ = 4.86 eV) than the triazine-cored analogs (Δ*E*_g_ = 4.33–4.38 eV). Within each series, the increase from six to nine peripheral carborane cages exerts only a small effect on the computed gap. A set of conceptual DFT reactivity descriptors, global hardness (*η*), electronegativity (*χ*), electrophilicity index (*ω*), and nucleophilicity index (*N*) was derived from the computed HOMO and LUMO energies according to standard relations^48,49^ (Table 3). Across the series, the triazine-cored compounds consistently exhibit higher electronegativity (*χ* = 4.20–4.59 eV) and substantially higher electrophilicity indices (*ω* = 4.47–4.82 eV) than the phenylene analogs (*χ* = 3.46–3.77 eV; *ω* = 2.45–2.92 eV), consistent with the electron-deficient character of the triazine core. Within each series, the nine-carborane derivative shows modestly higher electrophilicity than its six-carborane counterpart.

**Table 3:** Conceptual DFT reactivity descriptors of the four compounds, derived from the computed HOMO and LUMO energies at the B3LYP/6-31G level. Values illustrate qualitative trends across the series; absolute magnitudes are model-dependent

Compounds	Global hardness (*η*)	Electronegativity (*χ*)	Global electrophilicity index (*ω*)	Global nucleophilicity index (*N*)
Ph-6-CB	2.4328	3.4559	2.4543	0.4074
Ph-9-CB	2.4276	3.7677	2.9228	0.3421
Tz-6-CB	2.1663	4.2041	4.4725	0.2235
Tz-9-CB	2.1916	4.5949	4.8158	0.2076

Of the four compounds, Tz-9-CB is computationally identified as the most electrophilic and most electron-deficient member of the series (highest *ω*, lowest *N*). This qualitative ordering parallels the experimentally observed cytotoxicity trend, in which Tz-9-CB exhibits the lowest IC_50_ and highest selectivity index against MDA-MB-231 cells (Section 2.4). This is consistent with the expectation that more electrophilic dendritic frameworks engage more readily with nucleophilic biomolecular targets.^50^ We note this correspondence as a qualitative structure-property-activity trend across the series, rather than a quantitative predictor. The complete set of conceptual DFT descriptors (including *I*, *A*, *µ*, and *S*) is provided in the SI (Table S1).

#### 2.3.2 Qualitative TD-DFT analysis of excited States

TD-DFT calculations at the B3LYP/6-31G level with the integral-equation-formalism polarizable continuum model (IEF-PCM; acetone, *ε* = 20.493) were performed to qualitatively characterize the dominant low-lying vertical electronic transitions of the four compounds. Linear-response TD-DFT with global hybrid functionals such as B3LYP, combined with a non-polarized basis set and without excited-state geometry relaxation, is well known to misplace charge-transfer excitation energies and to distort oscillator strengths for systems of this type. Under these conditions, only the qualitative character of the transitions (orbital composition, relative oscillator strength) can be reliably extracted.

For both Ph-6-CB and Ph-9-CB, the lowest bright excitation (S_2_) arises primarily from HOMO → LUMO and HOMO → LUMO +1 transitions, with the transition density distributed largely within the central aromatic framework. This is consistent with π–π* character localized on the phenylene-substituted phenylene core. For Tz-6-CB and Tz-9-CB, the lowest bright excitation (also S_2_/S_3_) similarly arises from HOMO → LUMO and HOMO → LUMO +1 transitions, but the underlying orbital separation involves greater displacement of electron density from the aromatic core toward the carborane periphery, consistent with stronger ICT character. The qualitative distinction between the two series—phenylene: π–π* localized on the aromatic core; triazine: enhanced core-to-periphery ICT character—is the principal qualitative conclusion drawn from the TD-DFT results. The two lowest excited states of each compound are summarized in Table 4.

**Table 4:** Computed vertical excitation energies and orbital character of the two lowest singlet excited states of Ph-6-CB, Ph-9-CB, Tz-6-CB, and Tz-9-CB at the TD-B3LYP/6-31G level with IEF-PCM (acetone). Values are presented for qualitative comparison of relative excitation character within the series. Quantitative comparison with the experimental absorption maxima is not claimed and would not be supported by the present level of theory. Higher singlet states (S_3_–S_5_) are tabulated in SI (Table S2)

Molecules	S_*n*_	Energy (eV)	λ (nm)	f	Dominant transition
Ph-6-CB	S_1_	4.1690	297.40	0.0101	H → L
	S_2_	4.4954	275.80	0.9739	H → L, H → L + 1
Ph-9-CB	S_1_	4.1904	295.88	0.0077	H → L, H → L + 1
	S_2_	4.5086	274.99	0.9606	H → L, H → L + 1
Tz-6-CB	S_1_	3.6611	338.66	0.0000	H → L
	S_2_	3.8325	323.51	0.6296	H → L, H → L + 1
Tz-9-CB	S_1_	3.6723	337.62	0.0004	H → L
	S_2_	3.8514	321.92	0.8259	H − 1 → L

#### 2.3.3 Molecular electrostatic potential (MEP) analysis

Molecular electrostatic potential surfaces provide a qualitative visualization of regions of high and low electron density across the four dendritic frameworks, indicating likely sites of electrophilic and nucleophilic susceptibility. In conventional MEP color coding, red regions indicate high electron density (electrostatically negative, prone to electrophilic attack), blue regions indicate low electron density (electrostatically positive, prone to nucleophilic attack), and green regions are approximately neutral.

**Fig. 5:**
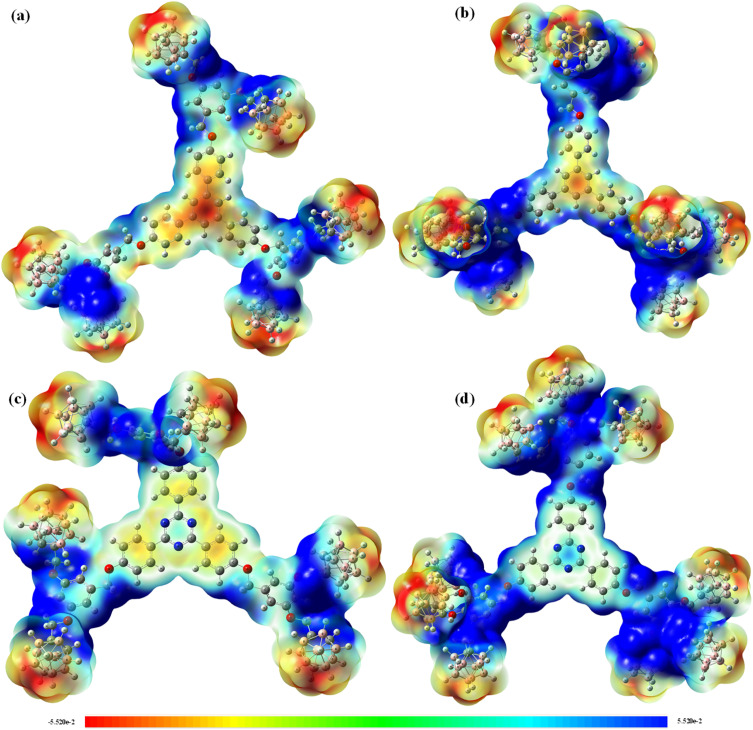
Molecular electrostatic potential (MEP) surface of the four synthesized carborane-containing dendritic compounds (a) Ph-6-CB, (b) Ph-9-CB, (c) Tz-6-CB and (d) Tz-9-CB computed at the B3LYP/6-31G level.

In all four compounds, the icosahedral *ortho*-carborane cages display a pronounced blue coloration, consistent with the σ-aromatic, electron-withdrawing character of the C_2_B_10_ cage (Fig. 5). The aromatic π-system and oxygen-containing ether linkers display yellow-to-red regions, indicating greater electron density at these positions. For the phenylene-cored compounds Ph-6-CB and Ph-9-CB, the central phenyl ring shows moderate orange coloration consistent with its near-neutral electronic character. For the triazine-cored analogs Tz-6-CB and Tz-9-CB, the triazine nitrogen atoms display localized blue regions reflecting the electron-poor character of the 1,3,5-triazine core, while the surrounding phenylene-oxygen linkers retain electron-rich (yellow-red) character. These qualitative observations are consistent with the FMO localization patterns discussed in Section 2.3.1.

### 2.4 Anti-cancer evaluation

#### 2.4.1 *In vitro* cytotoxicity assay

The cytotoxicity of the four synthesized carborane-containing dendritic compounds Ph-6-CB, Ph-9-CB, Tz-6-CB, and Tz-9-CB, was evaluated against the MDA-MB-231 triple-negative breast cancer cell and the non-cancerous NIH/3T3 mouse embryo fibroblast cell line using the MTT assay in DMSO. The half-maximal inhibitory concentration (IC_50_) values were calculated using MedCalc software and are presented in Table 5. The selectivity index, which measures the compounds' selectivity toward cancer cells, was calculated by taking the ratio of the IC_50_ obtained from healthy cells and cancerous cells.

**Table 5:** IC_50_ values (in µM) of Ph-6-CB, Ph-9-CB, Tz-6-CB and Tz-9-CB. ^a^Selectivity Index (SI) value is expressed as the ratio IC_50_ (NIH/3T3)/IC50 (MDA-MB-231), and the data were presented as mean values derived from three separate experiments conducted in triplicate

Compounds	IC_50_ value (in µM)		Selectivity index (SI)^a^
	NIH/3T3 (mouse embryo fibroblast cell line)	MDA-MB-231 (triple-negative breast cancer cell line)	
Ph-6-CB	90	32	2.81
Ph-9-CB	93	22	4.23
Tz-6-CB	82	18	4.56
Tz-9-CB	78	6	13
Cisplatin	67	56	1.20

**Fig. 6:**
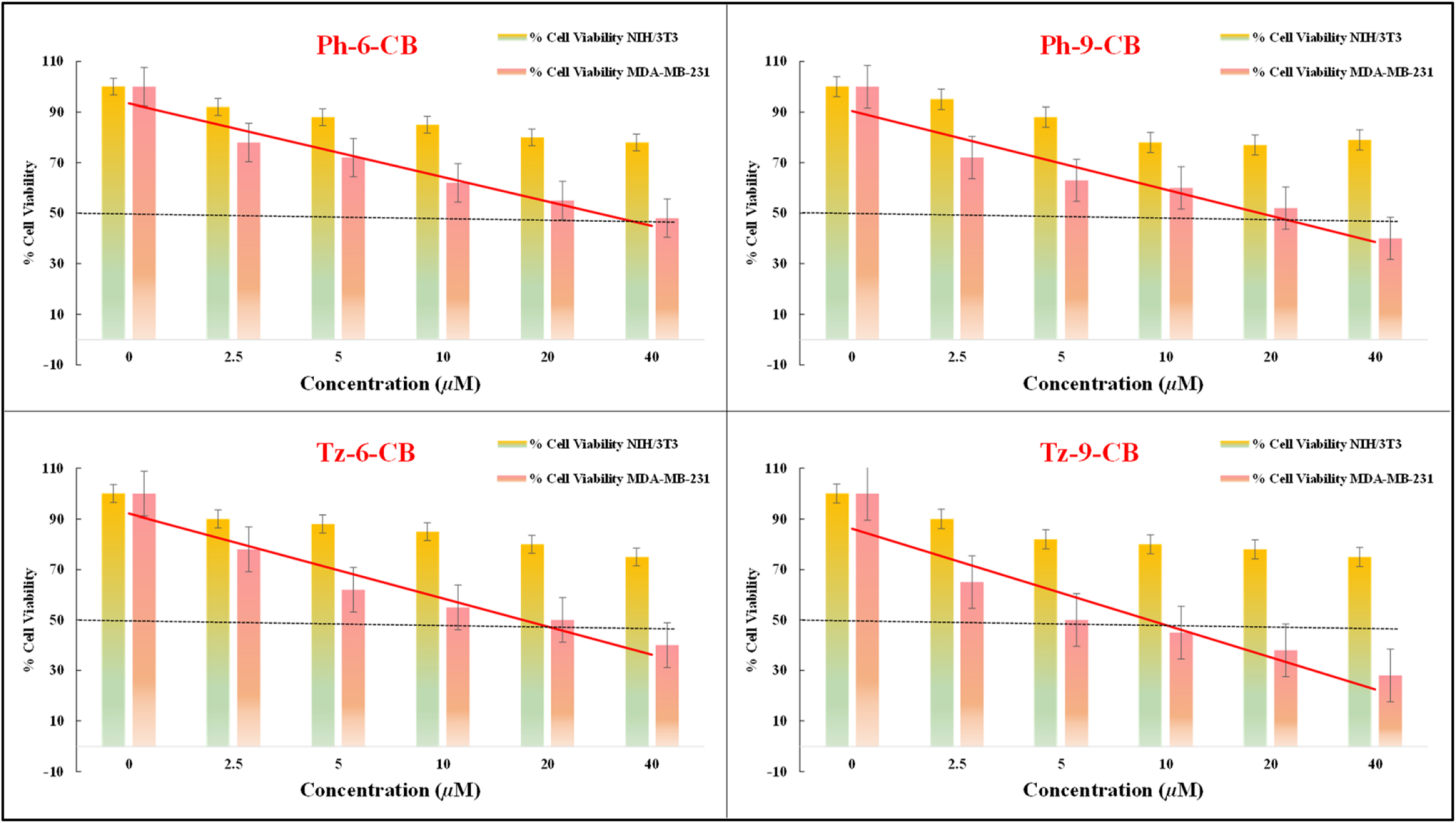
*In vitro* cytotoxic effect of Ph-6-CB, Ph-9-CB, Tz-6-CB and Tz-9-CB, at different concentrations (µM) on NIH/3T3 (Mouse embryo fibroblast cell line) and MDA-MB-231 (Triple-negative breast cancer cell line) determined using MTT assay (data are expressed as mean ± SD, *n* = 10 with respect to control).

All four compounds exhibited clear dose-dependent cytotoxicity toward the cancer cell line, whereas their toxicity toward NIH/3T3 cells remained comparatively low (Fig. 6). Among the phenyl-based systems, Ph-6-CB reduced cell viability with an IC_50_ of 32 µM, whereas Ph-9-CB, with a higher number of carborane units, exhibited improved potency (IC_50_ = 22 µM) and enhanced selectivity (SI = 4.23). This suggests that increasing the number of *ortho*-carborane cage in the phenylene-cored scaffold positively contributes to anticancer activity.

A more pronounced effect was observed in the triazine-cored derivatives. Tz-6-CB demonstrated stronger cytotoxicity toward MDA-MB-231 cells (IC_50_ = 18 µM) with an SI of 4.56. Remarkably, Tz-9-CB emerged as the most potent compound in the series, achieving 50% inhibition at a concentration of only 6 µM, while remaining significantly less toxic to normal fibroblasts (IC_50_ = 78 µM). The resulting selectivity index of 13 is more than ten-fold higher than that of cisplatin (SI = 1.20), highlighting the exceptional cancer-specific cytotoxicity of this compound.

A direct comparison with cisplatin underscores the advantage of the carborane-functionalized architectures. While cisplatin had an IC_50_ of 56 µM against MDA-MB-231 cells and exhibited limited selectivity for normal cells, all four *ortho*-carborane-based derivatives showed greater potency and markedly improved cancer selectivity. These findings reveal a clear structure-activity relationship wherein the triazine scaffold enhances anticancer activity more effectively than the phenyl framework, and an increased number of *o*-carborane units significantly boosts cytotoxic performance and specificity.

#### 2.4.2 Live-dead imaging

To further validate the cytotoxic effects observed in the MTT assay and to visually assess membrane integrity after treatment, live/dead imaging was performed using Hoechst 33342 and propidium iodide (PI) dual staining. Hoechst selectively stains the nuclei of all cells, emitting blue fluorescence, while PI penetrates only cells with compromised membranes, producing a red fluorescent signal indicative of non-viable or late-apoptotic cells. Thus, the combination of Hoechst-PI staining provides a reliable morphological readout to distinguish healthy, apoptotic, and dead cells based on nuclear condensation and membrane permeability.

**Fig. 7:**
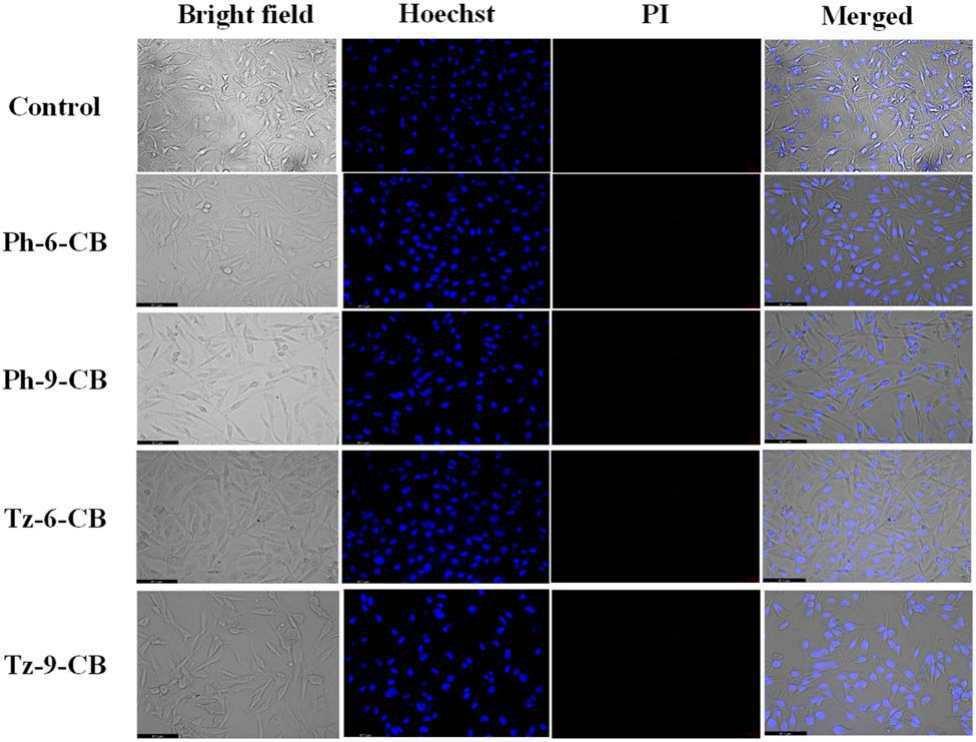
Bright-field and live/dead fluorescent images of NIH/3T3 cells treated with the IC_50_ concentrations of Ph-6-CB, Ph-9-CB, Tz-6-CB and Tz-9-CB. Cells were stained with Hoechst-PI to show viability after 48 h.

NIH/3T3 cells treated with the IC_50_ concentrations of carborane-containing dendritic compounds Ph-6-CB, Ph-9-CB, Tz-6-CB, and Tz-9-CB displayed intact nuclear morphology with minimal or no PI staining (Fig. 7). The cells retained their characteristic elongated fibroblast-like structure with uniformly dispersed Hoechst-stained nuclei, similar to the untreated control. The absence of significant red fluorescence indicates negligible membrane damage in normal fibroblasts, confirming the compounds' low cytotoxicity toward healthy cells even at their respective IC_50_ values. These visual observations strongly correlate with the high IC_50_ values obtained for NIH/3T3 cells in the MTT assay.

**Fig. 8:**
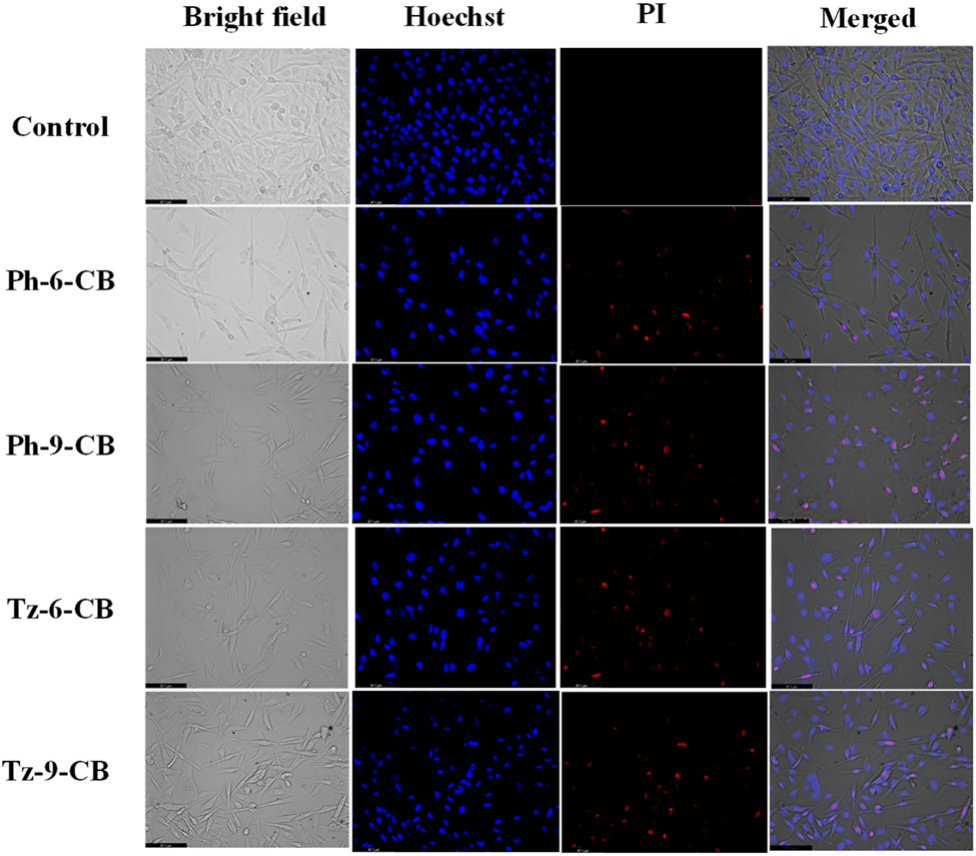
Bright-field and live/dead fluorescent images of MDA-MB-231 cells treated with the IC_50_ concentrations of Ph-6-CB, Ph-9-CB, Tz-6-CB and Tz-9-CB. Cells were stained with Hoechst-PI to show viability after 48 h.

In contrast, MDA-MB-231 cells treated with the same IC_50_ doses exhibited pronounced red PI fluorescence (Fig. 8), signifying compromised membrane integrity and extensive loss of cell viability. The number and intensity of PI-positive cells increased progressively in the order Ph-6-CB < Ph-9-CB < Tz-6-CB < Tz-9-CB, aligning well with their corresponding cytotoxicity profiles. Tz-9-CB, the most potent derivative, induced the greatest extent of nuclear condensation and PI uptake, indicating substantial apoptotic and necrotic cell populations. These morphological features are consistent with the markedly low IC_50_ value and high selectivity index of Tz-9-CB.

The live/dead imaging results visually corroborate the quantitative cytotoxicity data. All four carborane-containing compounds exhibit minimal toxicity toward normal fibroblasts while inducing significant cell death in the breast cancer cell line, particularly in the triazine-based derivatives. The high PI uptake in MDA-MB-231 cells confirms that the reduced viability observed in the MTT assay results from membrane-compromising cytotoxic effects, thereby validating the anticancer potential of these compounds.

#### 2.4.3 Caspase-3 activity study

A critical objective in the development of anticancer therapeutics is not only to demonstrate cytotoxicity but also to elucidate the specific mechanism by which cell death is induced. A key and highly desirable mechanism is apoptosis, or programmed cell death, which allows for the safe and controlled elimination of malignant cells without causing an inflammatory response. Central to this process is the caspase family of cysteine proteases, which orchestrate the apoptotic cascade. Among these, caspase-3 is considered a principal executioner caspase.^51,52^ Its activation represents a pivotal, often irreversible step that commits the cell to apoptosis by cleaving a host of vital cellular proteins. Therefore, measuring the activity of caspase-3 is a definitive method to confirm that a compound's cytotoxic effects are mediated through the induction of apoptosis, providing crucial insight into its mechanism of action and validating its potential as a targeted therapeutic agent.

**Fig. 9:**
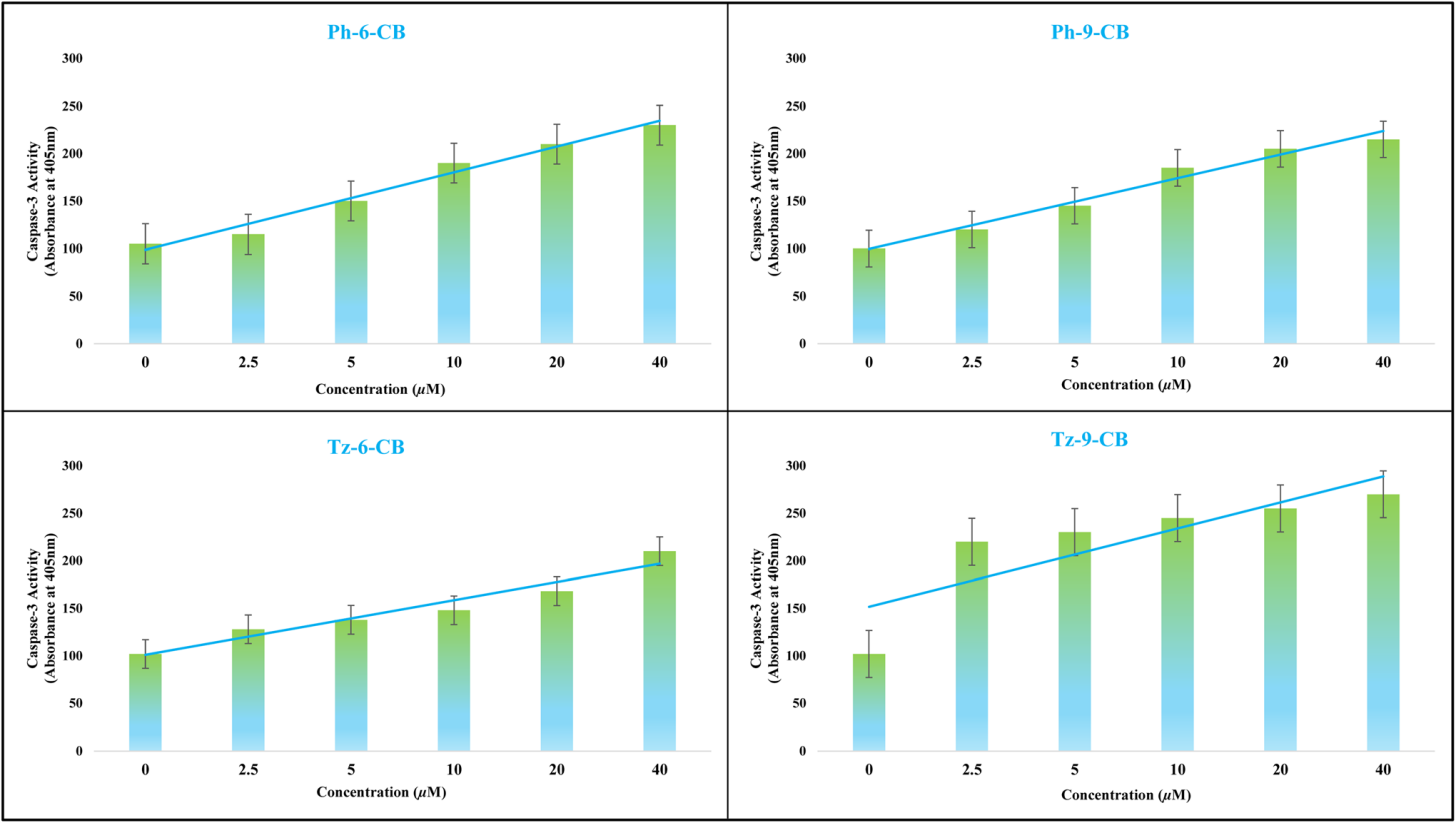
Caspase-3 activity of synthesized Ph-6-CB, Ph-9-CB, Tz-6-CB and Tz-9-CB at different concentrations (in µM) in MDA-MB-231 cancer cells.

To elucidate whether the decrease in cell viability induced by the carborane-containing dendrimers is associated with apoptosis, caspase-3 activity was quantified in MDA-MB-231 cells following treatment with carborane-containing dendritic compounds Ph-6-CB, Ph-9-CB, Tz-6-CB, and Tz-9-CB at different concentrations. As shown in Fig. 9, all four compounds induced a clear, concentration-dependent elevation in caspase-3 activity compared to the untreated control (set as 100% basal activity). Even at the lowest tested concentration, a noticeable increase in caspase-3 activity was observed, which progressively intensified with increasing dose. The phenyl-based derivatives Ph-6-CB and Ph-9-CB produced a moderate but consistent enhancement of caspase-3 activity, reaching approximately two-fold activation at the highest concentration tested. In contrast, the triazine-centered analogues Tz-6-CB and Tz-9-CB triggered a more pronounced response, with caspase-3 activity rising to nearly 2–2.5-fold relative to control at 40 µM. Among all four dendrimers, Tz-9-CB exhibited the strongest activation of caspase-3 across the entire concentration range, in line with its superior antiproliferative potency and highest selectivity index.

Taken together, these findings confirm that the carborane-appended dendrimers induce apoptosis in MDA-MB-231 cells *via* activation of the caspase-3 pathway and further support the designation of the triazine-based system, particularly Tz-9-CB, as the most effective pro-apoptotic candidate in this series.

## 3. Conclusion

In this work, four new carborane-appended symmetrical trimers, Ph-6-CB, Ph-9-CB, Tz-6-CB, and Tz-9-CB were synthesized, and evaluated to elucidate how central core electronics (phenylene *vs.* triazine) and peripheral *ortho*-carborane density (six *vs.* nine cages) collectively influence their photophysical, electronic, and biological properties.

Photophysical analysis revealed marked core-dependent differences. All four trimers absorb strongly in the 328–335 nm region, but phenylene-based systems showed higher absorption intensity compared to the triazine analogues. Emission characteristics were similarly core-modulated. Phenylene dendrimers retained higher fluorescence efficiencies, while triazine increased nonradiative decay, most prominently in Tz-9-CB. All compounds displayed large Stokes shifts (92–99 nm), indicating significant excited-state relaxation, with the largest shift observed for Tz-9-CB.

Qualitative DFT analysis supports the experimental observations in terms of trends across the series. HOMO localization on the central aromatic framework and LUMO localization on the peripheral *ortho*-carborane cages are consistent across all four compounds. The triazine-cored compounds exhibit qualitatively smaller HOMO–LUMO separations and substantially higher computed electrophilicity indices than the phenylene analogues, consistent with the electron-deficient character of the 1,3,5-triazine core.

The biological evaluation further highlighted the functional consequences of these electronic differences. All four trimers exhibited selective cytotoxicity toward MDA-MB-231 breast cancer cells while remaining significantly less toxic to NIH/3T3 fibroblasts. Phenylene-based derivatives showed IC_50_ values of 32 µM (Ph-6-CB) and 22 µM (Ph-9-CB) with selectivity indices of 2.81 and 4.23, respectively. In contrast, triazine derivatives displayed superior potency, with IC_50_ values of 18 µM (Tz-6-CB) and an exceptionally low 6 µM for Tz-9-CB, along with a remarkable selectivity index of 13. Live/dead imaging confirmed minimal membrane disruption in healthy cells but extensive PI uptake in cancer cells, particularly for Tz-9-CB. Mechanistic assays revealed dose-dependent caspase-3 activation, with Tz-9-CB producing the highest apoptotic response (up to 2.5-fold increase), correlating strongly with its cytotoxicity profile.

Overall, this study demonstrates a clear structure-property-function relationship:

• Phenylene cores support stronger conjugation, higher fluorescence, and moderate cytotoxicity.

• Triazine cores display lower quantum yield, narrower band gaps, higher electrophilicity, and significantly stronger, apoptosis-driven anticancer activity.

• Increasing *ortho*-carborane density from six to nine cages further amplifies these effects, with Tz-9-CB emerging as the most potent and selective compound in the series.

These findings provide valuable guidelines for designing next-generation *ortho*-carborane-based dendritic architectures with enhanced therapeutic potential.

## 4. Experimental

### 4.1 Materials and methods

The chemicals utilized in this study were obtained from Sigma-Aldrich/Merck India Pvt. Ltd/Spectrochem India and were used without further purification. The experimental procedures were conducted using oven-dried glassware in a dry argon/nitrogen environment. Solvents were distilled under a nitrogen atmosphere by using an appropriate drying agent. The reagents were purchased and utilized without additional purification. The purification of all the compounds was carried out by using silica gel column chromatography (60–120 mesh, Spectrochem, India). JEOL 400 MHz NMR spectrometer (JNM-ECZ400 s) was utilized to record the ^1^H, ^13^C, and ^11^B NMR spectra. Chemical shifts were reported with respect to tetramethylsilane (TMS) (^1^H: *δ* = 0.00 ppm) and CDCl_3_ (^13^C: *δ* = 77.0 ppm) as the reference compound and the coupling constants are given in Hz. All ^13^C spectra are proton-decoupled. ^11^B NMR spectra are proton-decoupled and were recorded at 128 MHz relative to BF_3_·Et_2_O. The Infrared (IR) spectra of all compounds were recorded on a Thermo Scientific Nicolet FT-IR spectrophotometer. Mass spectral analyses of newly synthesized compounds were carried out using HRMS and MALDI-TOF mass spectrometers. All the UV-vis absorption spectra were recorded using a Varian Cary 4000 UV-vis-NIR spectrophotometer with spectroscopic grade solvents. The emission spectra were recorded on a Fluoromax spectrofluorometer at room temperature in spectroscopic-grade solvents. The melting points of the compounds were determined using a conventional apparatus, and the data were uncorrected.

### 4.2 Computational study

All quantum chemical calculations were performed using the Gaussian 16 software package. Ground-state geometry optimizations of Ph-6-CB, Ph-9-CB, Tz-6-CB, and Tz-9-CB were carried out using density functional theory with the B3LYP hybrid exchange–correlation functional and the 6-31G basis set, without symmetry constraints.^53,54^ SCF convergence was set to 10^−8^ a.u., and the default ultrafine integration grid (99 radial shells, 590 angular points) was employed throughout. Harmonic vibrational frequency analyses were performed at the same level of theory; all optimized geometries were confirmed as true minima on the potential energy surface, with no imaginary frequencies.

Vertical electronic excitation energies, wavelengths, and oscillator strengths for the lowest singlet excited states were obtained from linear-response TD-DFT calculations^55,56^ at the same level of theory (B3LYP/6-31G), with solvent effects included *via* the integral-equation-formalism polarizable continuum model^57,58^ (IEF-PCM) using acetone (*ε* = 20.493) as the implicit solvent to match the experimental measurement conditions. Excited-state geometry relaxation was not performed; only vertical Franck–Condon excitations are reported. Frontier molecular orbital (FMO) analysis, conceptual DFT reactivity descriptors, and molecular electrostatic potential (MEP) surfaces were derived from the optimized ground-state wavefunctions using standard relations. All visualizations were prepared in GaussView 6.0.

Methodological note: the B3LYP/6-31G level of theory employed in this work is acknowledged to be an approximate model for boron-rich dendritic systems and for excitations with significant charge-transfer character. Accordingly, the computational results presented in Sections 2.3.1–2.3.3 are interpreted qualitatively, and no quantitative comparison between computed and experimental absorption maxima is undertaken. A quantitatively predictive treatment of these dendrimers would require, consistent with current best practice in carborane computational chemistry,^46^ at minimum: (i) polarized triple-zeta or larger basis sets (*e.g.*, 6-311G*, def2-TZVP), in place of the unpolarized 6-31G basis set used here; (ii) a long-range-corrected or modern *meta*-GGA exchange–correlation functional suitable for charge-transfer states (*e.g.*, CAM-B3LYP, *ω*B97X-D); (iii) conformational sampling *via* classical or QM/MM molecular dynamics, followed by clustering and local QM minimization; and (iv) relaxation of the lowest excited-state geometries. These elements form the basis of ongoing follow-up computational work on this dendrimer family.

### 4.3 Cell culture

Dulbecco's Modified Eagle Medium (DMEM), High glucose, and fetal bovine serum (FBS) were purchased from HiMedia chemicals. 0.25% Trypsin-EDTA was procured from Thermo Fisher scientific. Penicillin-Streptomycin, 3-(4,5-dimethylthiazol-2-yl)-2,5-diphenyltetrazolium bromide (MTT), Hoechst 33342, propidium iodide (PI), dimethyl sulfoxide (DMSO) was purchased from Sigma-Aldrich Chemicals Company. Phosphate buffered saline (PBS) was purchased from SRL Chemicals. All chemicals for cell culture were purchased from PAN Biotech (GmbH, Germany) unless otherwise mentioned. All the required reagents and solutions were prepared using autoclaved double distilled water (DDW). Human Triple Negative Breast Adenocarcinomas cell line MDA-MB-231 and Mouse embryo fibroblast cell line NIH/3T3 were obtained from the National Center for Cell Science, Pune, India. Cell lines were properly maintained in Dulbeccos Minimal Essential Medium (DMEM) supplemented with 10% Foetal bovine serum (FBS), 1% l-Glutamine, and 1% Penicillin- Streptomycin. The cell lines were maintained at 37 °C in a 5% CO_2_/95% relative humidity in the CO_2_ incubator (Memmert India laboratory).

### 4.4 MTT assay

MDA-MB 231 and NIH/3T3 cell lines were maintained in a DMEM culture medium supplemented with 10% FBS. When the cells reached the logarithmic growth phase, they were seeded in 96-well culture plates at an optimal density (5 × 10^3^ cells per well) in a CO_2_ (5%) incubator at 37 °C. After 24-hour incubation period, the culture medium was removed. Triplicate wells were treated with different concentrations of the *ortho*-carborane appended symmetrical trimers (Ph-6-CB, Ph-9-CB, Tz-6-CB, and Tz-9-CB). Cellular responses were examined with the MTT assay by measuring absorbance at 570 nm after 48 h, and cell morphology was analyzed by microscopy on day 2. Cells cultured in medium alone served as the control. Experiments were performed in triplicate. Cell growth curves were calculated as the mean values of each group. This treatment dose was used for all further cell culture experiments. The cell viability percentage was calculated using the standardized formula:
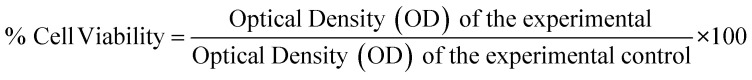


### 4.5 Live-dead imaging

The cell viability was examined by Hoechst 33342/propidium iodide (PI) double fluorescence staining. MDA-MB 231 and NIH/3T3 cells at the logarithmic growth stage were cultured in 6-well plates and were treated with obtained IC_50_ concentrations of the *ortho*-carborane appended star-shaped molecules (Ph-6-CB, Ph-9-CB, Tz-6-CB, and Tz-9-CB) after 48 h. Thereafter, the cells were stained with 10 µL Hoechst 33342 solution and 5 µL PI in the dark at 25 °C for 10 min each. Staining results were observed using Leica Microsystems CMS GmbH DMi8 inverted fluorescence microscope, and images were collected.

### 4.6 Preparation and analytical data of compounds

#### 4.6.1 Compound 5a

Methyl-3,5-dihydroxybenzoate 1a (10.0 g, 59.471 mmol) was dissolved in 200 mL of dry acetone. To this solution, potassium carbonate (36.11 g, 261.67 mmol) and propargyl bromide (80% in toluene, 19.5 mL) were added. The resulting mixture was refluxed at 80 °C for 20 hours, filtered through a silica pad, and subsequently concentrated to obtain compound 2a in good yield. Compound 2a was utilized in further reactions without purification. LiAlH_4_ (6.21 g, 163.76 mmol) was suspended in dry THF (200 mL) and cooled to 0 °C. A solution of compound 2a (10.0 g, 40.94 mmol) in 30 mL of dry THF was then added to it at 0 °C. The mixture was stirred at 0 °C for 1 h and then at room temperature for 6 h. LiAlH_4_ was quenched with the dropwise addition of water very carefully, and then the reaction mixture was extracted with dichloromethane. The organic layer was washed with water, dried over anhydrous MgSO_4_, and evaporated to get 9.0 g of pure product compound 3a in good yield. Compound 3a was used for the next step without further purification. To the solution of compound 3a (9.0 g, 41.62 mmol) in dichloromethane (150 mL), PBr_3_ (4 mL, 41.62 mmol) was added, and the reaction mixture was stirred at room temperature for 15 h. After the reaction was over, water was added to the reaction mixture and extracted with dichloromethane. The organic layer was washed with water, dried over anhydrous MgSO_4_, and concentrated to get the compound 4a.^7,42^ Without further purification, compound 4a was used for the next reaction. Decaborane (745 mg, 6.101 mmol) was refluxed in 4 mL of dry acetonitrile for 2 h under an argon atmosphere until a yellow precipitate was formed. The reaction mixture was allowed to cool to room temperature, followed by the addition of alkynyl dendron 4a (600 mg, 2.033 mmol) in dry acetonitrile (5 mL). Again, it was refluxed at 90 °C for 12 h. After the completion of the reaction, the reaction mixture was cooled to room temperature, and methanol (10 ml) was added to quench the excess decaborane. Then the reaction mixture was evaporated using a rotary evaporator to obtain the crude mixture. The crude product was purified by silica gel chromatography using 6% ethyl acetate in hexane as eluent to obtain 335 mg of pure compound 5a as a colorless solid. Yield: 31%. Mp: 222 °C. ^1^H NMR (400 MHz, CDCl_3_, *δ* ppm): 6.54 (d, 2H, *J* = 2.4 Hz), 6.29 (t, 1H, *J* = 2.4 Hz), 4.39 (s, 4H, O–CH_2_), 4.37 (s, 2H, CH_2_–Br), 4.03 (s, 2H, C_Cage_-H). ^13^C NMR (100 MHz, CDCl_3,_*δ* ppm): 158.16, 140.96, 108.95, 102.10, 70.84 (C_cage_), 69.18 (C_cage_), 57.76, 32.20. ^11^B NMR (proton-decoupled, 128 MHz, CDCl_3_, *δ* ppm): −3.55, −5.27, −9.89, −12.54, −13.91. IR (KBr): 2593 (B–H), 2361, 2343, 1602, 1508, 1457, 1410, 1374, 1301, 1245 cm ^−1^. HRMS (*m*/*z*): calcd for C_13_H_31_B_20_BrO_2_: 517.9942; found: 540.5351[M + Na]^+^.

#### 4.6.2 Compound 5b

Methyl-3,4,5-trihydroxybenzoate 1b (2.0 g, 10.86 mmol) was dissolved in 50 mL of dry acetone. To this solution, potassium carbonate (8.9 g, 65.16 mmol) and propargyl bromide (80% in toluene, 3.2 mL) were added. The resulting mixture was refluxed at 80 °C for 20 hours, filtered over a silica pad, and concentrated to obtain compound 2b in 86% yield. LiAlH_4_ (1.21 g, 31.22 mmol) was suspended in dry THF (20 mL) and cooled to 0 °C. Then a solution of compound 2b (2.0 g, 7.80 mmol) in 5 mL of dry THF was added to it at 0 °C. The mixture was stirred at 0 °C for 1 h and then at room temperature for 6 h. The completion of the reaction was monitored using TLC. LiAlH_4_ was quenched with the dropwise addition of water very carefully, and then the reaction mixture was extracted with ethyl acetate. The organic layer was washed with water, dried over anhydrous Na_2_SO_4_, and evaporated to get 1.7 g of pure product 3b in 81% yield. To the solution of compound 3b (1.0 g, 3.69 mmol) in dichloromethane (40 mL), PBr_3_ (0.34 mL, 3.69 mmol) was added, and the reaction mixture was stirred at room temperature for 15 h. After the reaction was over, water was added to the reaction mixture, and the product was extracted with dichloromethane. The organic layer was washed with water, dried over anhydrous MgSO_4_, and concentrated to get the compound 4b. The product was used in the next steps without further purification. Decaborane (666 mg, 5.446 mmol) was refluxed in 4 mL of dry acetonitrile for 2 h under an argon atmosphere until a yellow precipitate was formed. The reaction mixture was allowed to cool to room temperature, followed by the addition of alkynyl dendron 4b (500 mg, 1.513 mmol) in dry acetonitrile (4 mL). Again, it was refluxed at 90 °C for 12 h. After the completion of the reaction, the reaction mixture was cooled to room temperature, and methanol (10 ml) was added to quench the excess decaborane. Then, the reaction mixture was evaporated using a rotary evaporator to obtain the crude mixture. The crude product was purified by silica gel chromatography using 16% ethyl acetate in hexane as eluent to obtain 390 mg of pure compound 5b as a colorless solid. Yield: 31%. Mp: 255 °C. ^1^H NMR (400 MHz, CDCl_3_, *δ* ppm): 6.54 (s, 2H), 4.42 (s, 4H, O–CH_2_), 4.35 (s, 2H, O–CH_2_), 4.30 (s, 2H, CH_2_-Br), 4.05 (s, 1H, Cage-H) 3.96 (s, 2H, Cage-H) ^13^C NMR (100 MHz, CDCl_3_, *δ* ppm): 150.76, 135.85, 134.78, 108.47, 73.13 (C_cage_), 71.19 (C_cage_), 70.73 (C_cage_), 70.30 (C_cage_), 58.62, 58.14, 32.12. ^11^B NMR (proton-decoupled, 128 MHz, CDCl_3_, *δ* ppm): −3.41, −5.10, −9.81, −12.59, −13.88. IR (KBr): 2593 (B–H), 2361, 2343, 1602, 1508, 1457, 1410, 1374, 1301, 1245 cm ^−1^. HRMS (*m/z*): calcd for C_16_H_43_B_30_BrO_3_: 686.5405; found: 709.5161[M + Na]^+^.

#### 4.6.3 Compound Tz-6-CB

2,4,6-Tris (*p*-hydroxyphenyl) triazine 7 (50 mg, 0.139 mmol) was solubilized in 10 mL of dry acetone.^42^ To this solution, *ortho*-carborane dendron 5a (266 mg, 0.50 mmol) and K_2_CO_3_ (173 mg, 1.257 mmol) were added, and the reaction mixture was refluxed at 60 °C for 16 h. The mixture was filtered through a silica bed, and the solvent was evaporated using a rotary evaporator. The residue was purified by silica gel column chromatography using 30% EtOAc in hexane as the eluent, affording 104 mg of pure compound Tz-6-CB as a colorless solid. Yield: 44%; mp: >250 °C. ^1^H NMR (400 MHz; DMSO-*d*_6_, *δ* ppm): 8.64 (d, 6H, *J* = 8.0 Hz, Ar–H), 7.20 (d, 6H, *J* = 8.0 Hz, Ar–H), 6.80 (d, 6H, *J* = 2.4 Hz, Ar–H), 6.64 (t, 3H, *J* = 4 Hz, Ar–H), 5.33 (s, 6H, Cage-H), 5.17 (s, 6H, OCH_2_-H), 4.62 (s, 12H, OCH_2_-H). ^13^C NMR (100 MHz, DMSO-*d*_6_, *δ* ppm): 170.08 (triazine ring-C), 162.00, 158.34, 139.45, 130.57, 128.22, 115.09, 107.84, 101.36, 73.42(C-cage), 68.62(C-cage), 61.88, 54.94.^11^B NMR(proton-decoupled, 128 MHz, DMSO-*d*_6_, *δ* ppm): −4.19, −10.44, −12.80. IR (KBr): 3081, 2931, 2565(B–H Streching), 2359, 1601, 1501, 1366, 1244, 1165, 1022, 808 cm^−1^.MALDI-TOF-MS (*m*/*z*): calcd for C_60_H_105_B_60_N_3_O_9_: 1661.1120; found: 1661.2662 [M]^+^.

#### 4.6.4 Compound Tz-9-CB

2,4,6-Tris (*p*-hydroxyphenyl) triazine 7 (40 mg, 0.116 mmol) was solubilized in 10 mL of dry acetone. To this solution, *ortho*-carborane dendron 5b (278 mg, 0.40 mmol) and K_2_CO_3_ (140 mg, 1.004 mmol) were added, and the reaction mixture was refluxed at 60 °C for 36 h. The mixture was filtered through a silica bed, and the solvent was evaporated using a rotary evaporator. The residue was purified by silica gel column chromatography using 80% EtOAc in hexane as the eluent, affording 65 mg of pure compound Tz-9-CB as a colorless solid. Yield: 33%; mp: >265 °C. ^1^H NMR (400 MHz, DMSO-*d*_6_, *δ* ppm): 8.63 (d, 6H, *J* = 8.0 Hz, Ar–H), 7.19 (d, 6H, *J* = 8.0 Hz, Ar–H), 6.98 (s, 6H, Ar–H), 6.56 (s, 6H, OCH_2_-H), 5.24 (s, 3H, Cage-H), 5.08 (s, 6H, Cage-H), 4.65 (s, 12H, OCH_2_-H), 4.44 (s, 6H, OCH_2_-H),. ^13^C NMR (100 MHz, DMSO-*d*_6_, *δ* ppm) 170.14 (triazine ring-C), 162.18, 151.25, 135.76, 132.51, 130.59, 128.19, 115.11, 107.83, 79.49 (C-cage), 79.11 (C-cage), 78.51 (C-cage), 78.01 (C-cage), 69.66, 59.47, 56.52. ^11^B NMR (proton-decoupled, 128 MHz, DMSO-*d*_6_, *δ* ppm):−4.29,−10.45,−13.93,−17.73. IR (KBr): 2916, 2854, 2591(B–H Streching), 1594, 1502, 1355, 1247, 1123, 1014, 814, 721 cm^−1^. MALDI-TOF-MS (*m*/*z*): calcd for C_69_H_141_B_90_N_3_O_12_: 2194.8890; found, 2194.4403 [M]^+^.

#### 4.6.5 Compound Ph-6-CB

2,4,6-Tris (*p*-hydroxyphenyl) benzene 9 (40 mg, 0.112 mmol) was solubilized in 10 mL of dry acetone.^7^ To this solution, *ortho-*carborane dendron 5a (216 mg, 0.406 mmol) and K_2_CO_3_ (140 mg, 1.008 mmol) were added, and the reaction mixture was refluxed at 60 °C for 16 h. The mixture was filtered through a silica bed, and the solvent was evaporated using a rotary evaporator. The residue was purified by silica gel column chromatography using 25% EtOAc in hexane as the eluent, affording 80 mg of pure compound Ph-6-CB as a colorless oily liquid. Yield: 42%. ^1^H NMR (400 MHz, CDCl_3_, *δ* ppm): 7.65 (s, 3H, Ar–H), 7.63 (d, 6H, *J* = 8.0 Hz, Ar–H), 7.05 (d, 6H, *J* = 8.0 Hz, Ar–H), 6.63 (d, 6H, *J* = 2 Hz, Ar–H), 6.33 (t, 3H, *J* = 2.0 Hz Ar–H), 5.05 (s, 6H, OCH_2_-H), 4.42 (s, 12H, OCH_2_-H), 4.05 (s, 6H, Cage-H). ^13^C NMR (100 MHz, CDCl_3_, *δ* ppm): 158.30, 157.97, 141.62, 140.55, 134.29, 128.41, 123.90, 115.11, 106.96, 101.57, 71.01 (Cage-C), 69.31(OCH_2_-C), 69.09(Cage-C), 57.79(OCH_2_-C).^11^B NMR (proton-decoupled, 128 MHz, CDCl_3_, *δ* ppm):-3.59, −4.75, −9.88, −12.50. IR (Neat): 3078, 2924, 2583(B–H Streching), 1594, 1502, 1447, 1378, 1161, 1014, 821, 737 cm^−1^. MALDI-TOF-MS (*m*/*z*): calcd for C_63_H_108_B_60_O_9_:1658.1480; found: 1658.4625 [M]^+^.

#### 4.6.6 Compound Ph-9-CB

2,4,6-Tris (*p*-hydroxyphenyl) benzene 9 (30 mg, 0.084 mmol) was solubilized in 10 mL of dry acetone. To this solution, *ortho-*carborane dendron 5b (210 mg, 0.304 mmol) and K_2_CO_3_ (106 mg, 0.761 mmol) were added, and the reaction mixture was refluxed at 60 °C for 36 hours. The mixture was filtered through a silica bed, and the solvent was evaporated using a rotary evaporator. The residue was purified by silica gel column chromatography using 55% EtOAc in hexane as the eluent, affording 67 mg of pure compound Ph-9-CB as a colorless oily liquid. Yield: 36%. ^1^H NMR (CDCl_3_, 400 MHz, *δ* ppm): 7.63 (s, 3H,Ar–H), 7.60 (d, 6H, *J* = 8.0 Hz, Ar–H), 7.01 (d, 6H, *J* = 8.0 Hz, Ar–H), 6.61 (s, 6H, Ar–H), 5.01 (s, 6H, OCH_2_-H), 4.44 (s, 12H, OCH_2_-H), 4.29 (s, 6H, OCH_2_-H), 4.09 (s, 3H, Cage-H), 3.96 (s, 6H, Cage-H). ^13^C NMR (100 MHz, CDCl_3_, *δ* ppm): 157.70, 151.03, 141.57, 135.20, 134.43, 128.45, 123.97, 115.11, 106.54, 105.68, 73.20 (C-cage), 71.17 (C-cage), 70.84 (C-cage), 70.27 (C-cage), 69.10, 58.51, 58.08. ^11^B NMR (proton-decoupled, 128 MHz, CDCl_3_, *δ* ppm): −3.30, −5.03, −9.71, −12.42, −13.57. IR (Neat): 3068, 2926, 2588(B–H Streching), 1589, 1508, 1440, 1373, 1224, 1015, 819, 711 cm^−1^. MALDI-TOF-MS (*m*/*z*): calcd, for C_72_H_144_B_90_O_12_: 2174.8320; found, 2174.7013 [M]^+^.

## Conflicts of interest

The authors declare no competing financial interests.

## Supplementary Material

RA-016-D6RA04231G-s001

RA-016-D6RA04231G-s002

## Data Availability

The data supporting this article have been included as part of the supplementary information (SI). Supplementary information is available. See DOI: https://doi.org/10.1039/d6ra04231g.
