# Supplementary material for: Synthesis, photophysical, computational, and cytotoxicity evaluation of carborane-appended phenylene and triazine trimers: elucidating the role of the central core
Source: RSC Adv. 2026 Jun 1;16(32):29733–50. doi: 10.1039/d6ra04231g (PMC13227497; doi:10.1039/d6ra04231g)
Supplement: RA-016-D6RA04231G-s002 [file RA-016-D6RA04231G-s002.pdf]

Supplementary Information (SI) for RSC Advances.
This journal is © The Royal Society of Chemistry 2026

Supporting Information

Synthesis, Photophysical, Computational, and Cytotoxicity Evaluation of Carborane-Appended Phenylene and Triazine Trimers: Elucidating the Role of the Central Core

Simran Pattnaik[a], Anwesha Pradhan[b], Laxmipriya Nayak[a], Subhadeep Acharya[a], Supriya Routray[a], Subhasri Dan[c], Soumya Ranjan Jena[b], Kshatresh Dutta Dubey[c], Luna Samanta[b], Rashmirekha Satapathy[a,*]

[a] *Department of Chemistry, Ravenshaw University, Cuttack-753003, Odisha, India*
[b] *Department of Zoology, Ravenshaw University, Cuttack-753003, Odisha, India*
[c] *Department of Chemistry, School of Natural Sciences, Shiv Nadar Institution of Eminence, Delhi NCR*
**Corresponding Author*
Email: rashmi@ravenshawuniversity.ac.in

Contents **Page No.**

NMR Spectra………………………………………………………………………. 2

Mass Spectra………................……………………………………………………. 11

Additional Computational Data... 15

NMR spectra

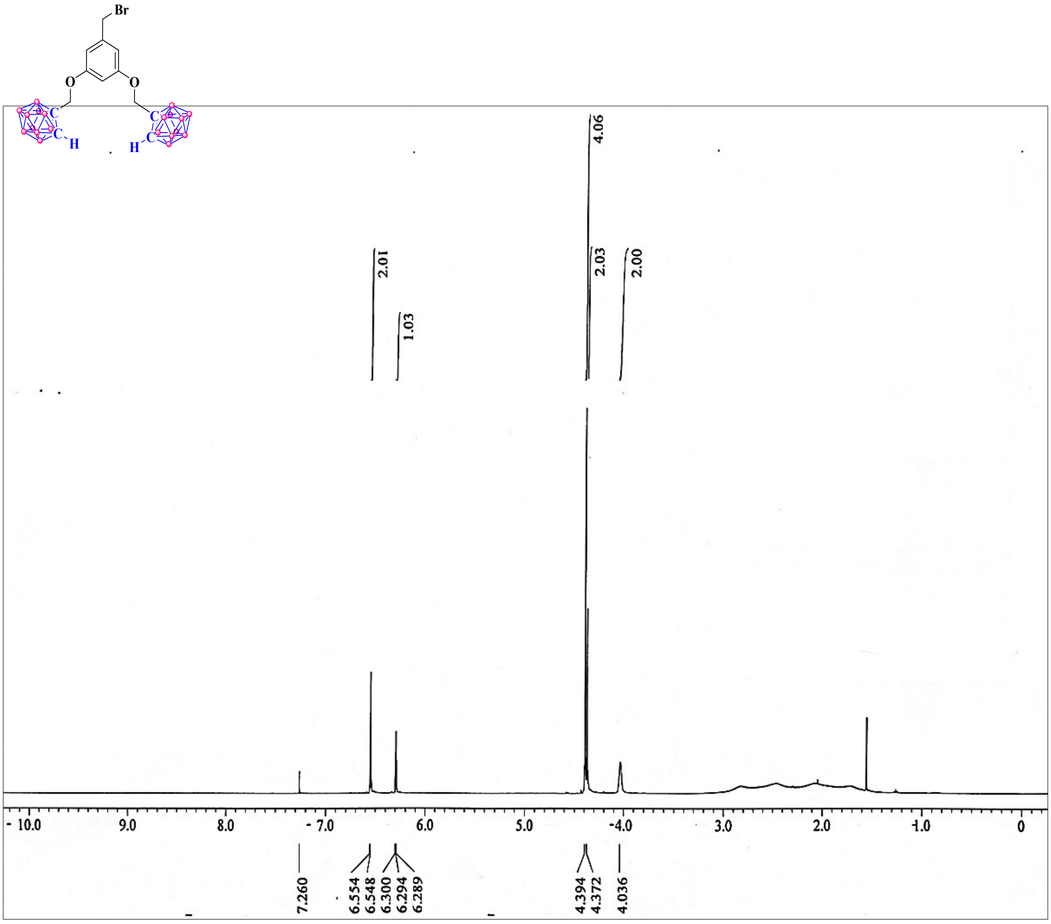

Figure S1: ^{1}H NMR of compound **5a**

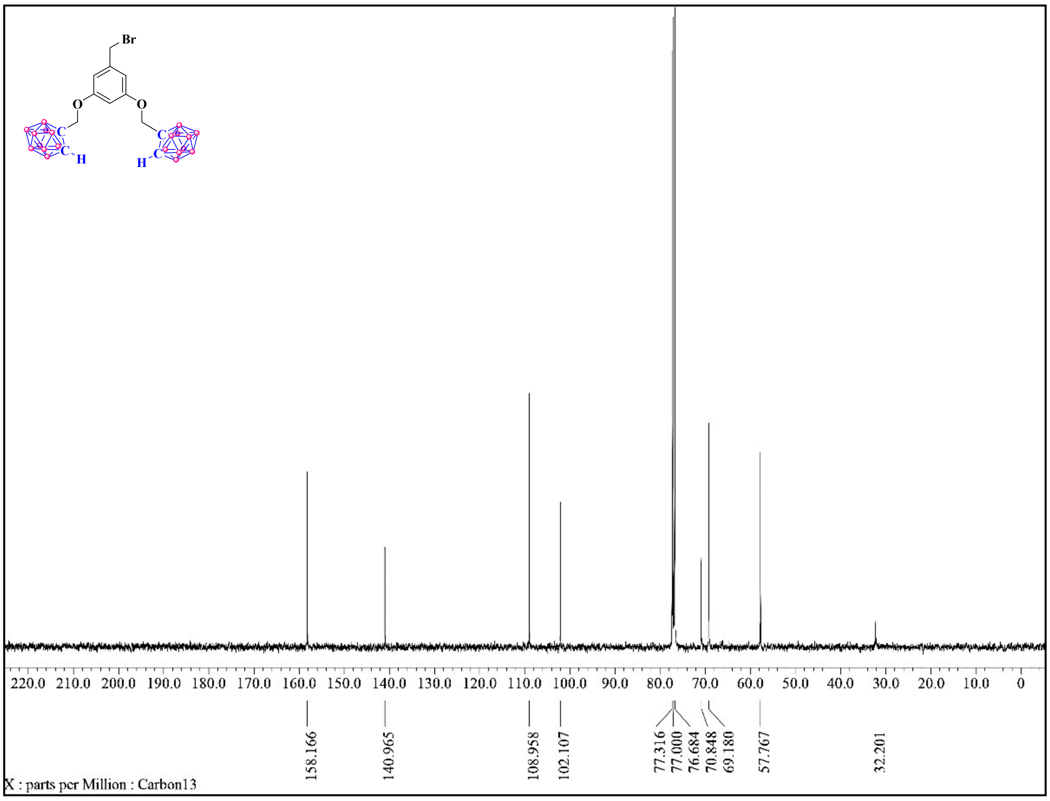

Figure S2: ^{13}C NMR of compound **5a**

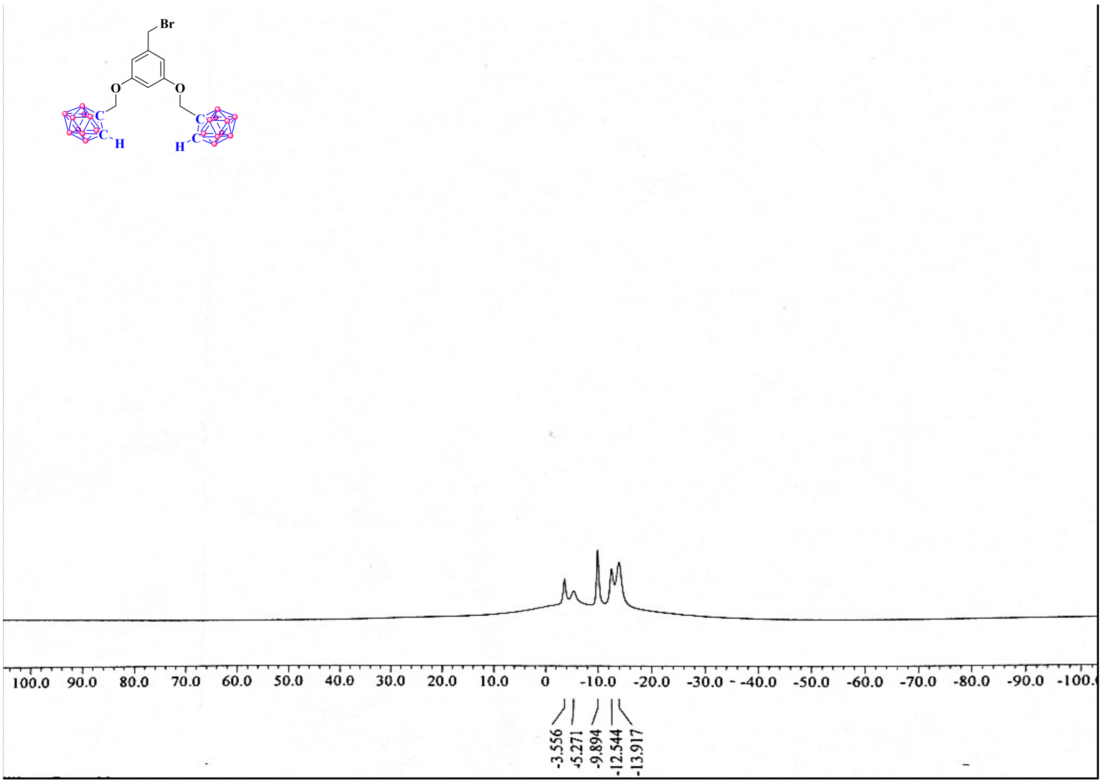

Figure S3: ^{11}B NMR of compound **5a**

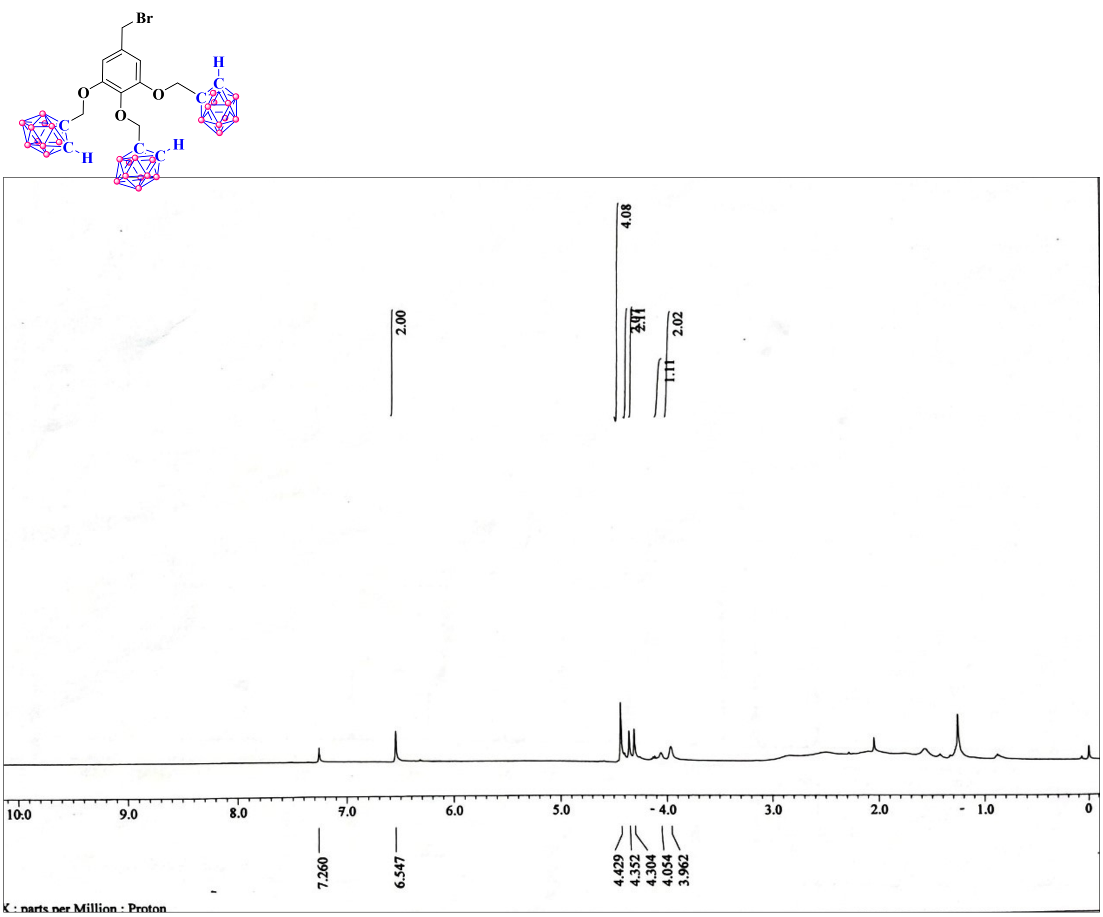

Figure S4: ^{1}H NMR of compound **5b.**

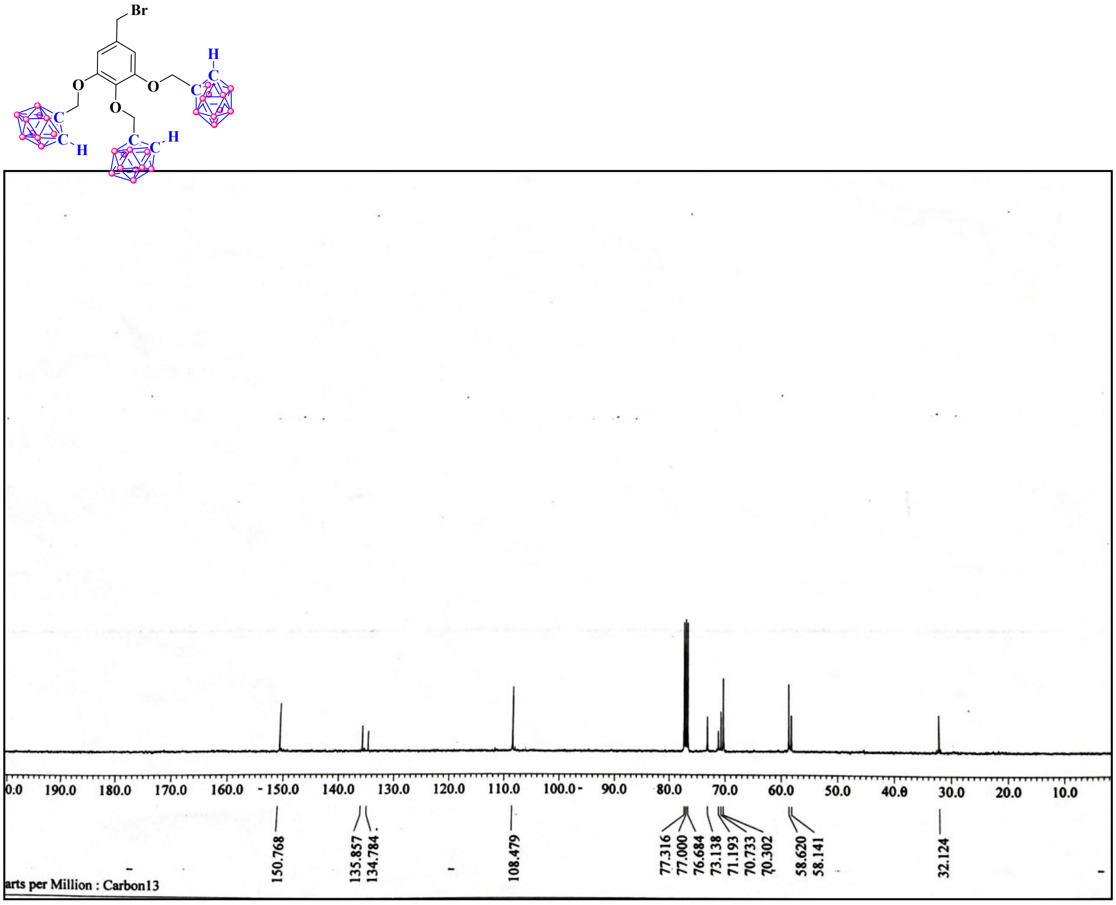

Figure S5: ^{13}C NMR of compound **5b**

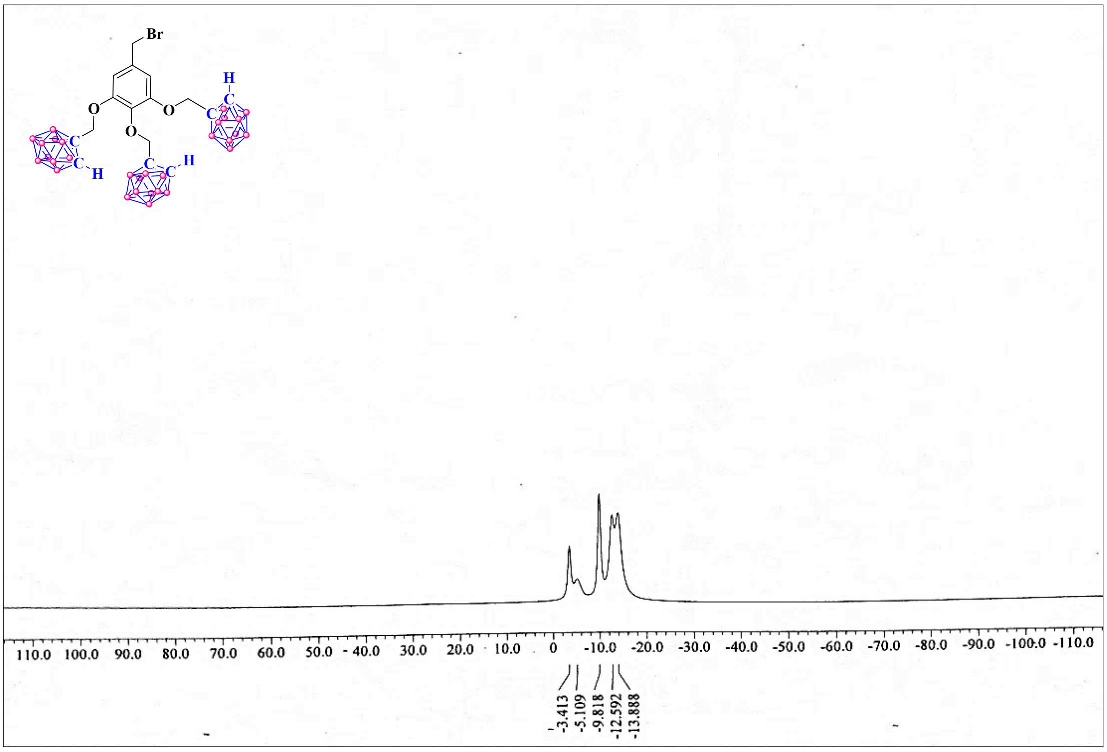

Figure S6: ^{11}B NMR of compound **5b**

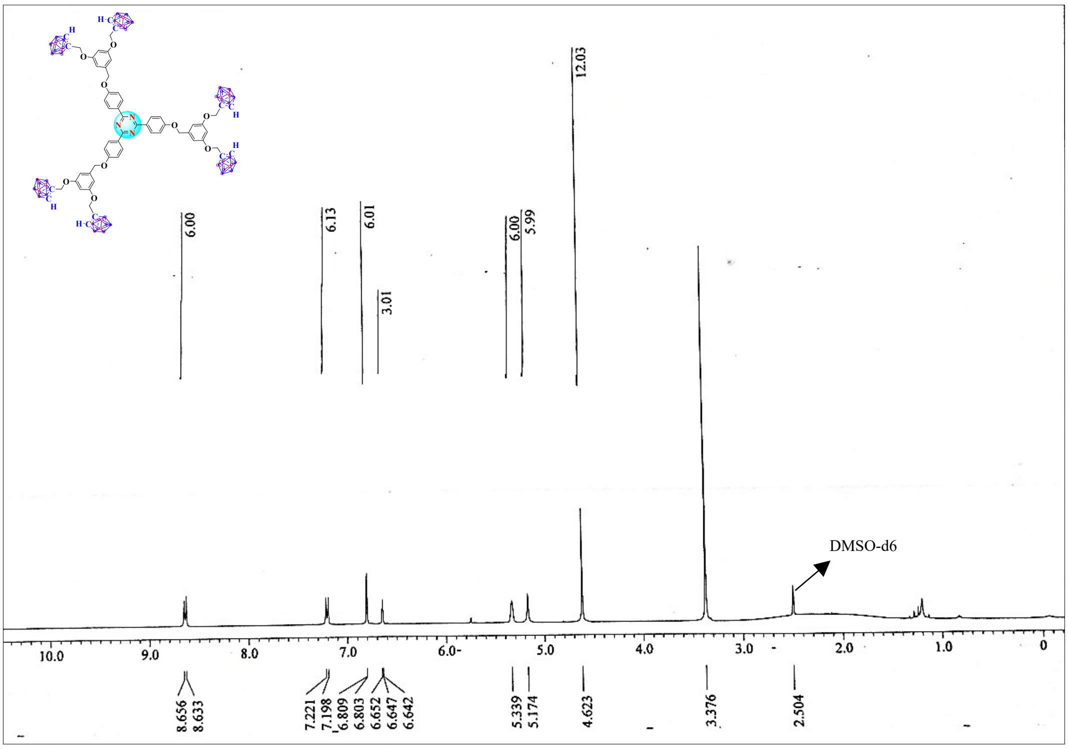

Figure S7: ^{1}H NMR of compound **Tz-6-CB.**

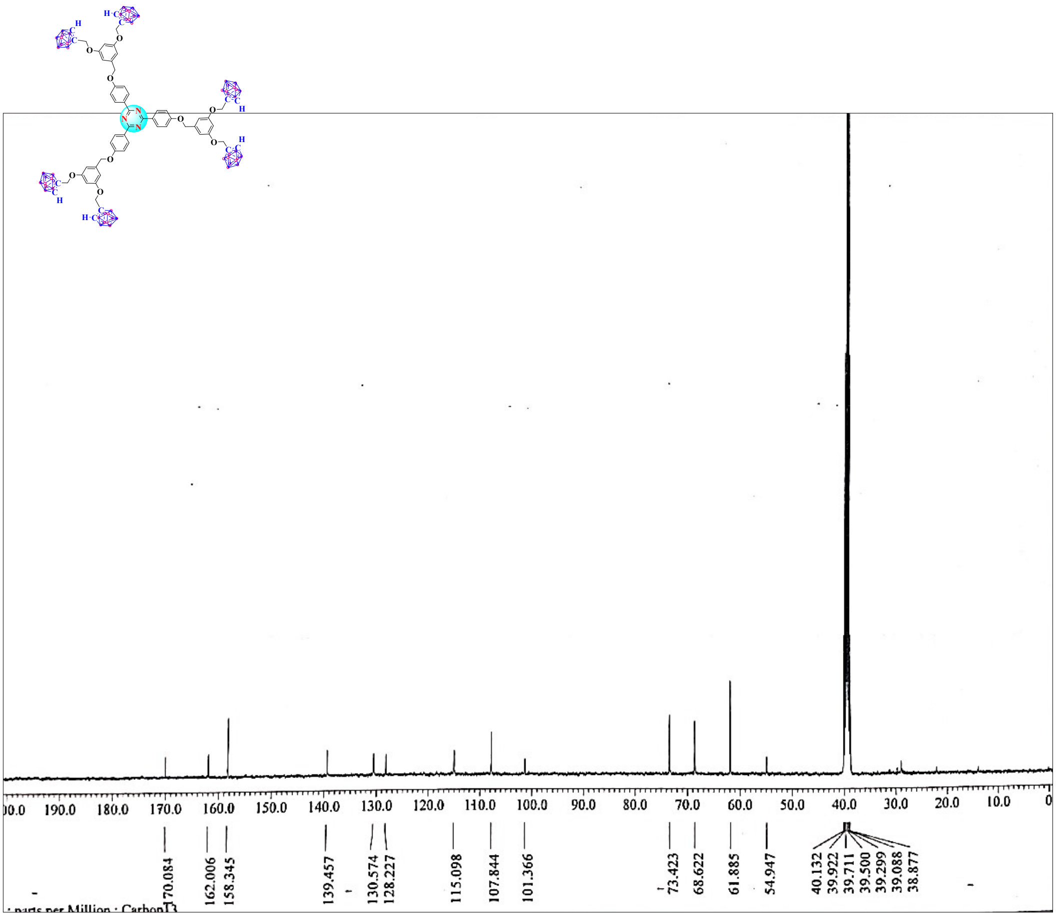

Figure S8: ^{13}C NMR of compound **Tz-6-CB.**

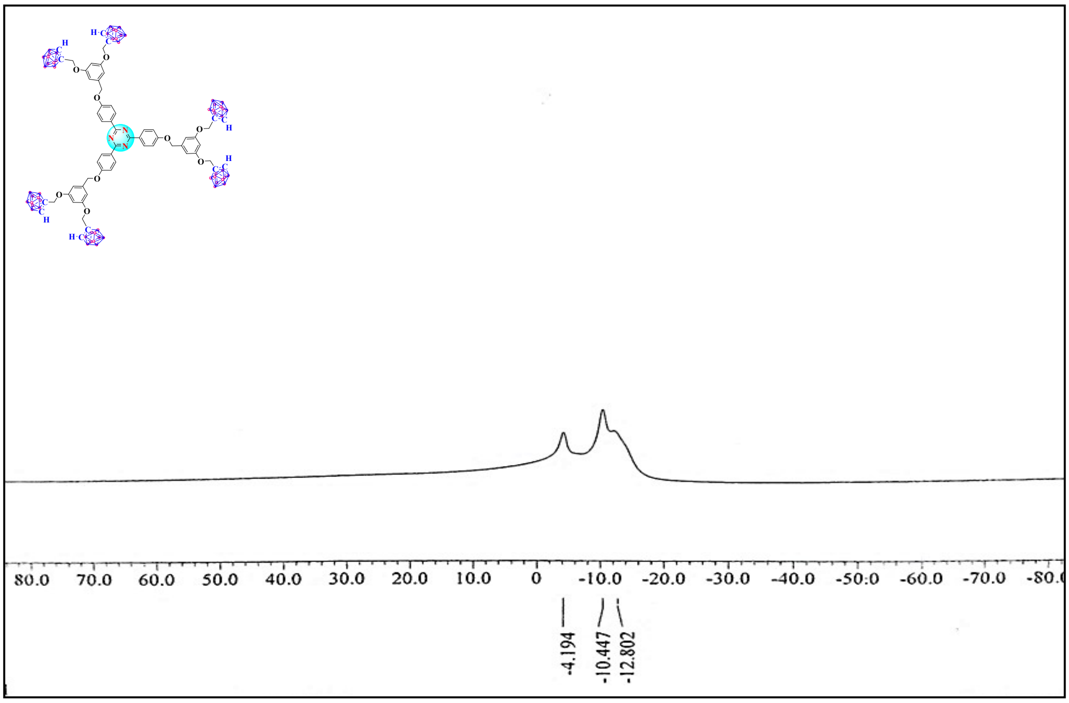

Figure S9: ^{11}B NMR of compound **Tz-6-CB.**

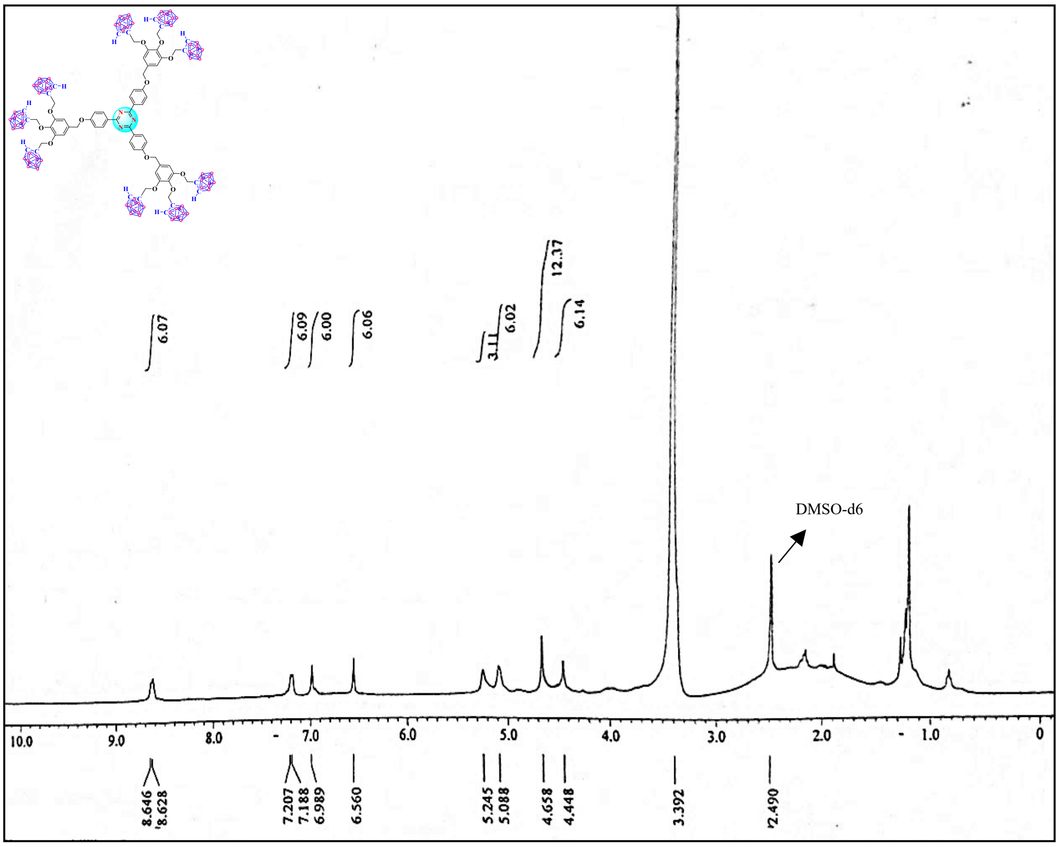

Figure S10: ^{1}H NMR of compound **Tz-9-CB.**

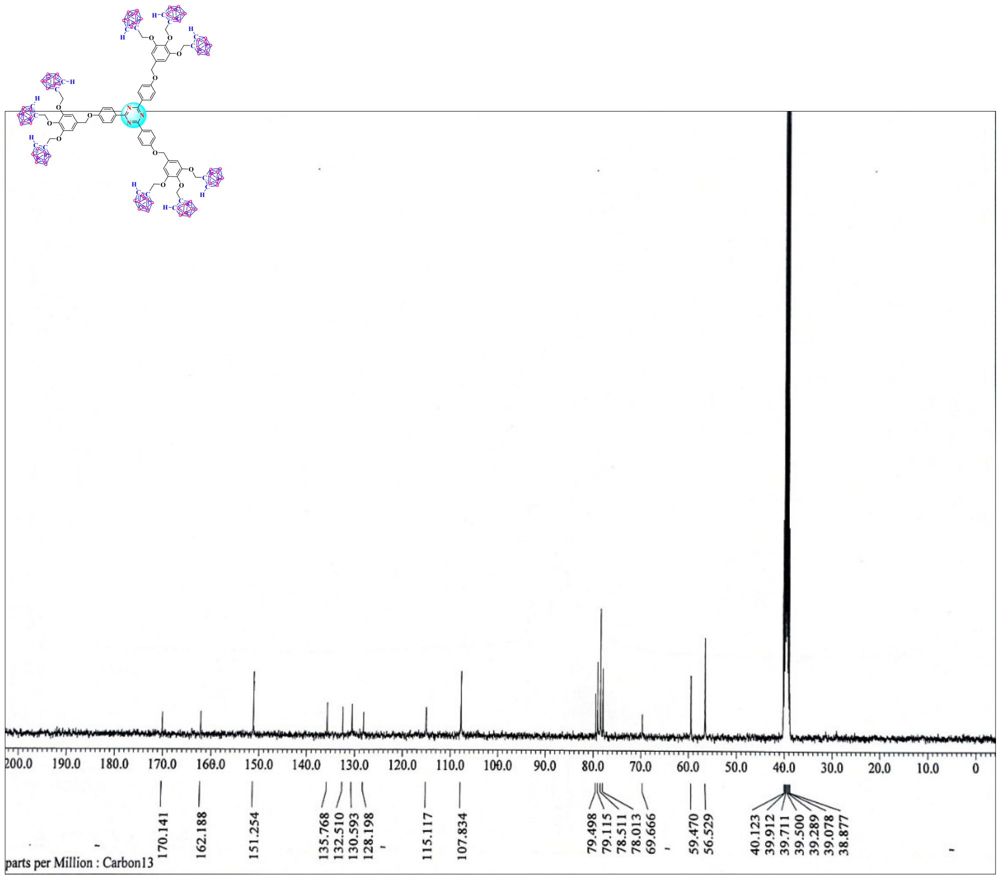

200.0
190.0
180.0
170.0
160.0
150.0
140.0
130.0
120.0
110.0
100.0
90.0
80.0
70.0
60.0
50.0
40.0
30.0
20.0
10.0
0
170.141
162.188
151.254
135.768
132.510
130.593
128.198
115.117
107.834
79.498
79.115
78.511
78.013
69.666
59.470
56.529
40.123
39.912
39.711
39.500
39.289
39.078
38.877
parts per Million : Carbon13

Figure S11: ^{13}C NMR of compound **Tz-9-CB.**

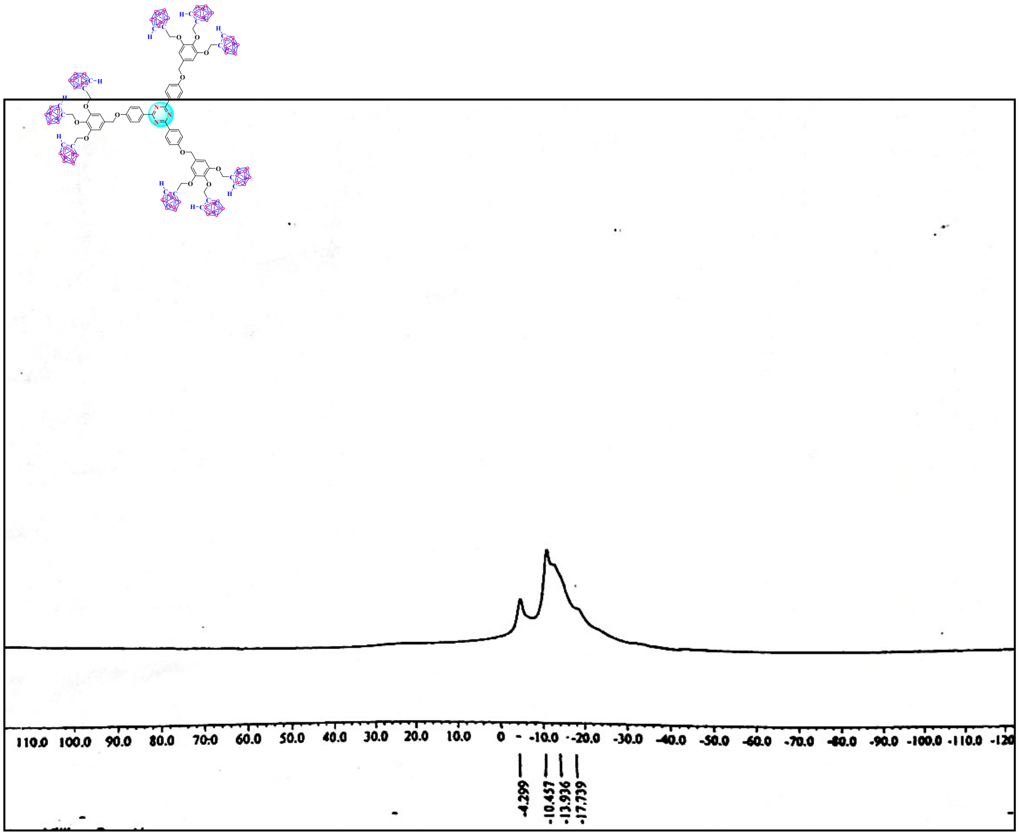

Figure S12: ^{11}B NMR of compound **Tz-9-CB**.

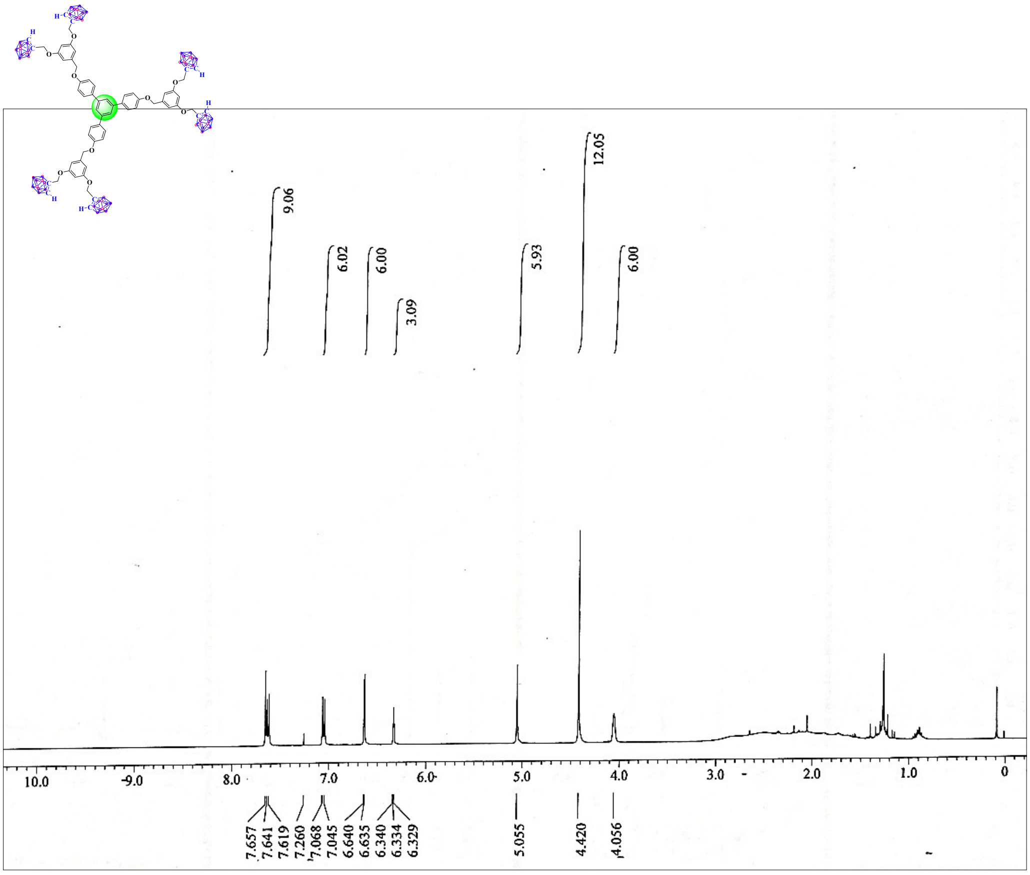

Figure S13: ^{1}H NMR of compound **Ph-6-CB.**

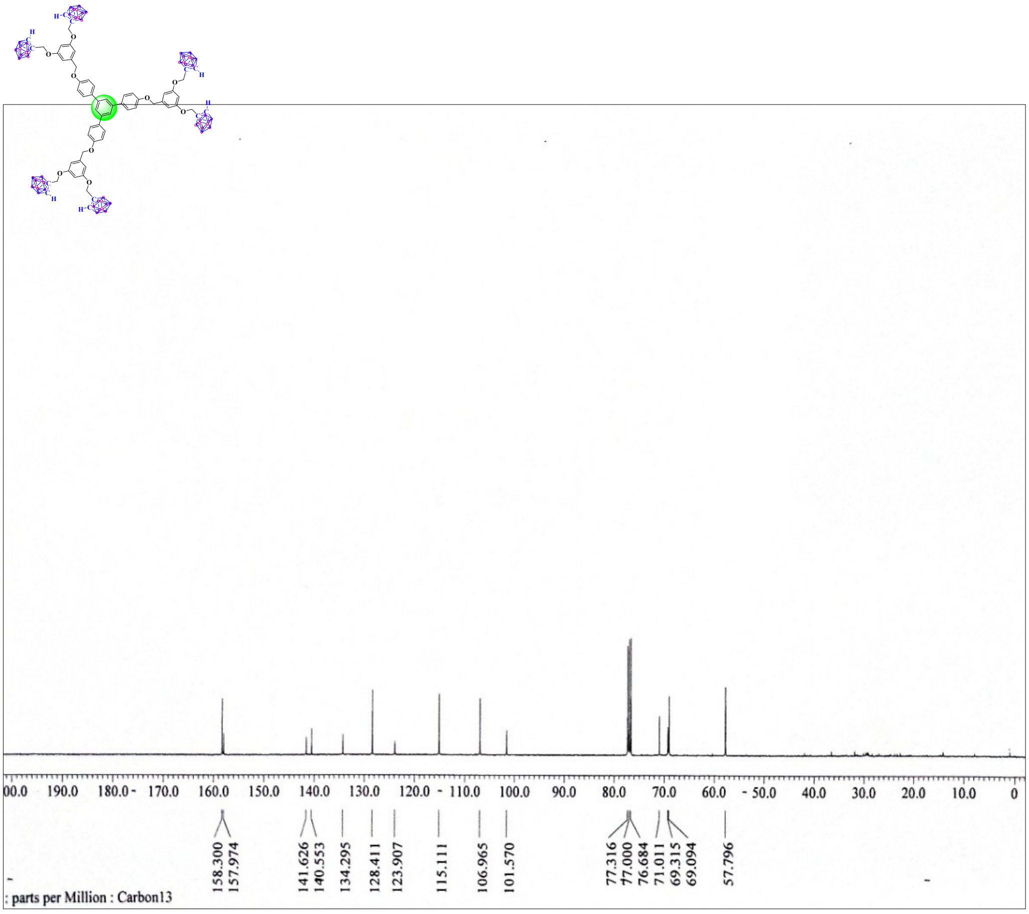

Figure S14: ^{13}C NMR of compound **Ph-6-CB.**

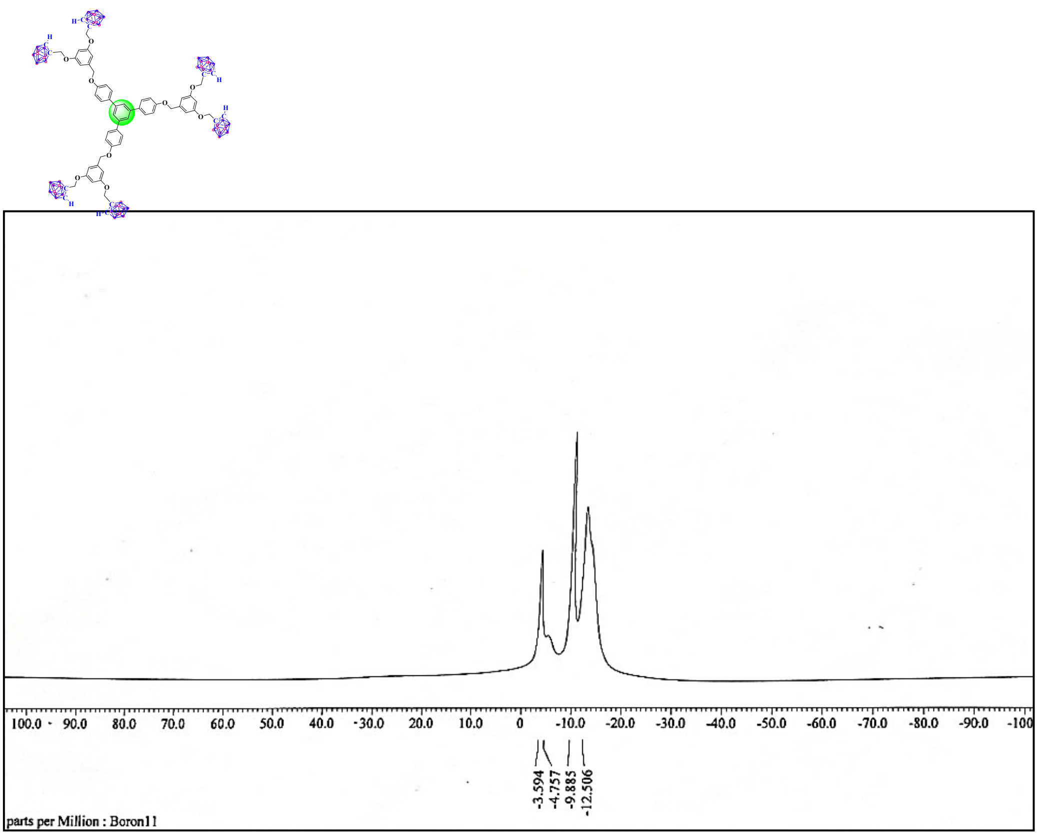

Figure S15: ^{11}B NMR of compound **Ph-6-CB.**

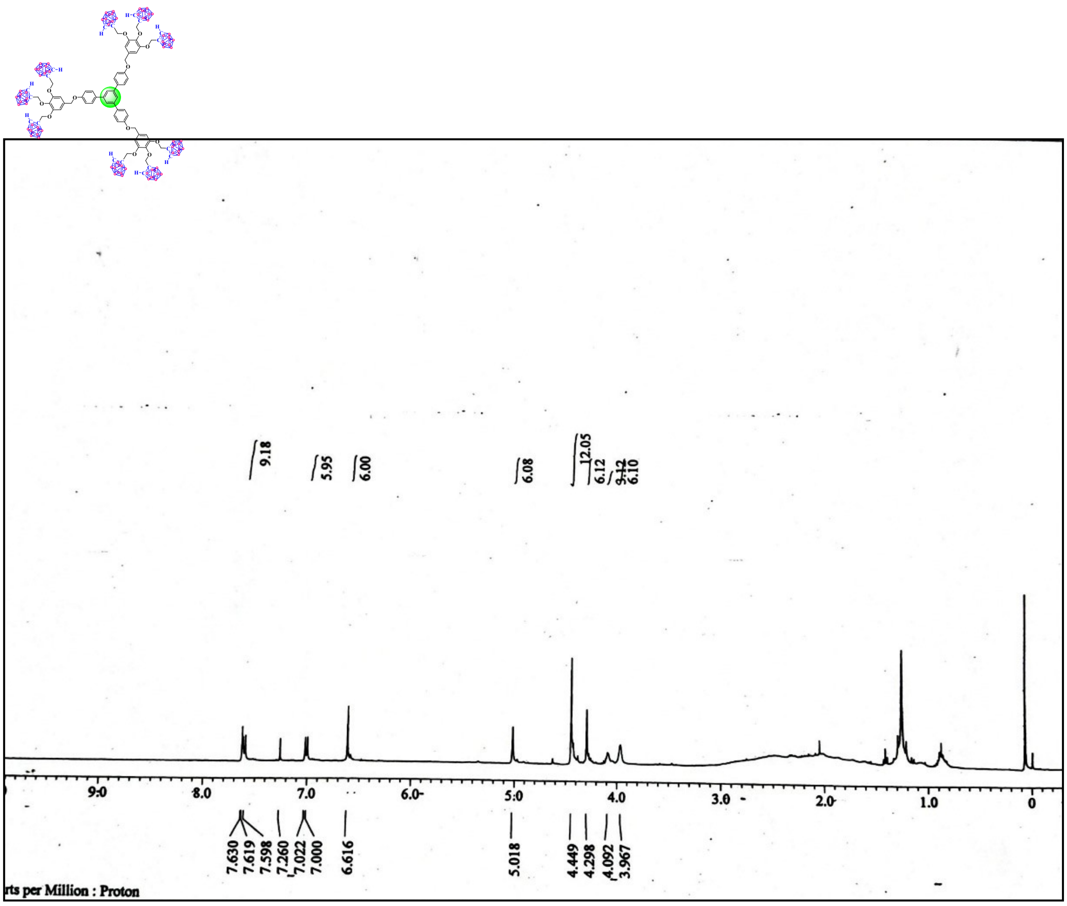

Figure S16: ^{1}H NMR of compound **Ph-9-CB.**

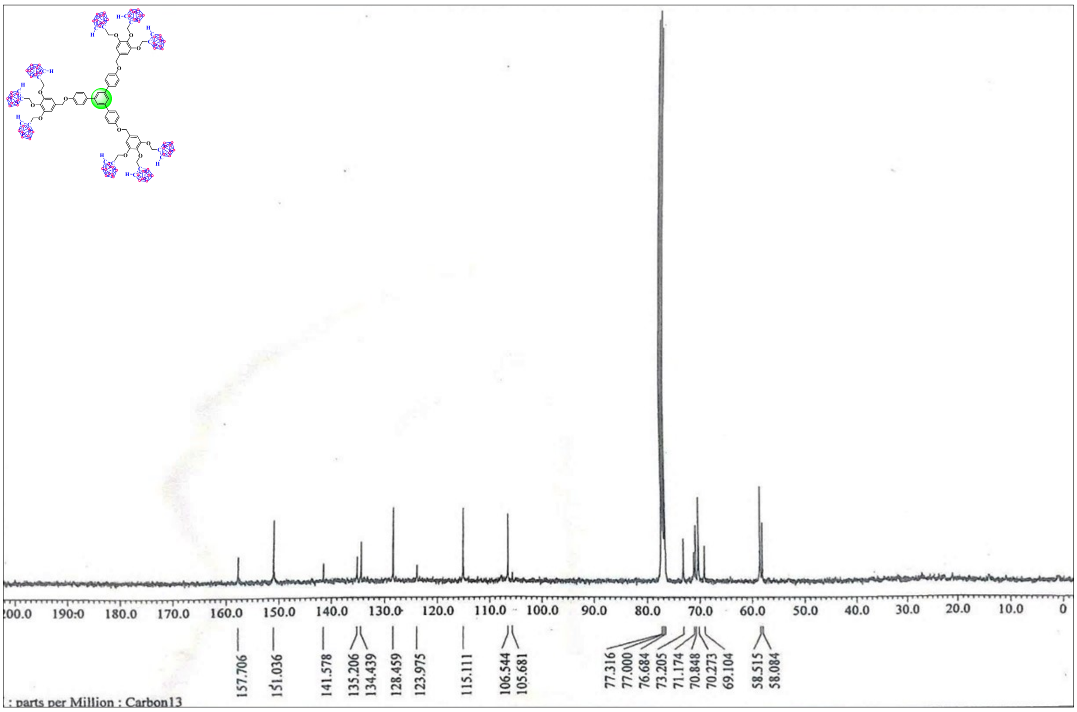

Figure S17: ^{13}C NMR of compound **Ph-9-CB.**

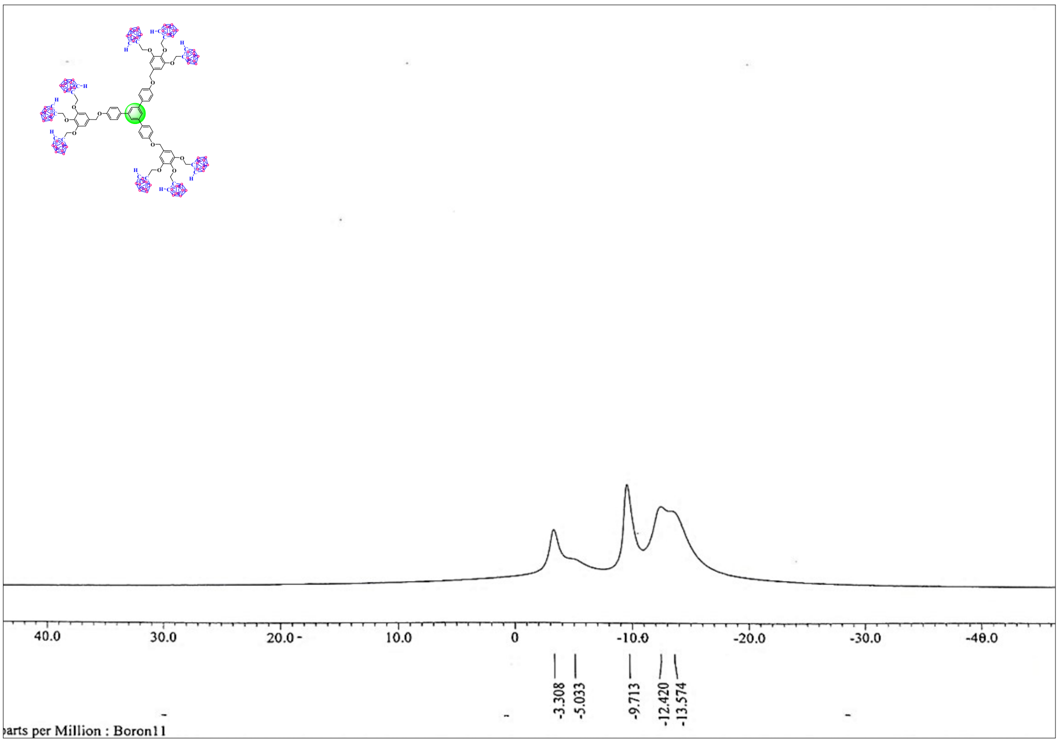

Figure S18: ^{11}B NMR of compound **Ph-9-CB.**

Mass Spectra

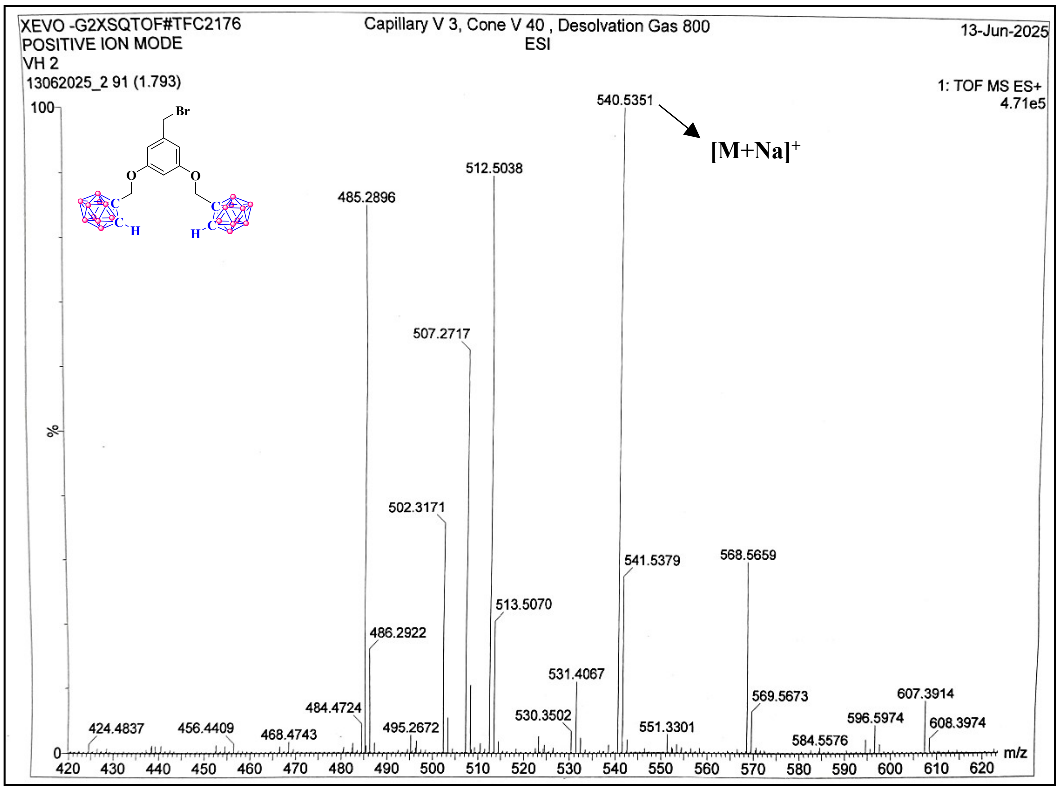

Figure S19: Mass spectra of compound **5a.**

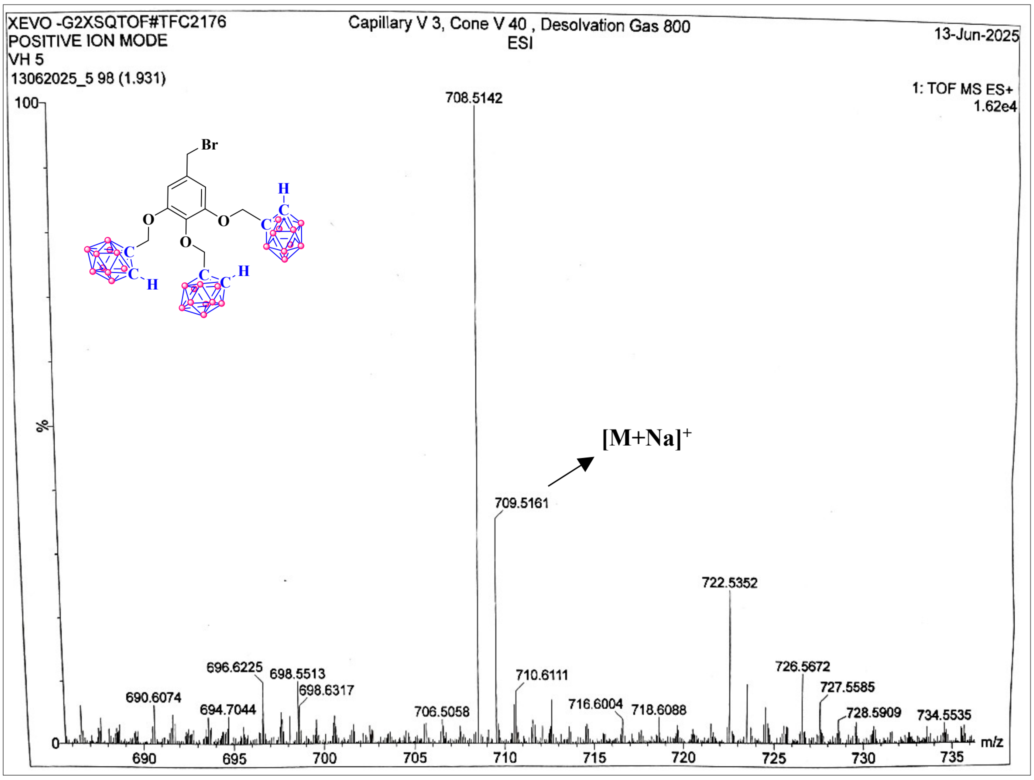

Figure S20: Mass spectra of compound **5b.**

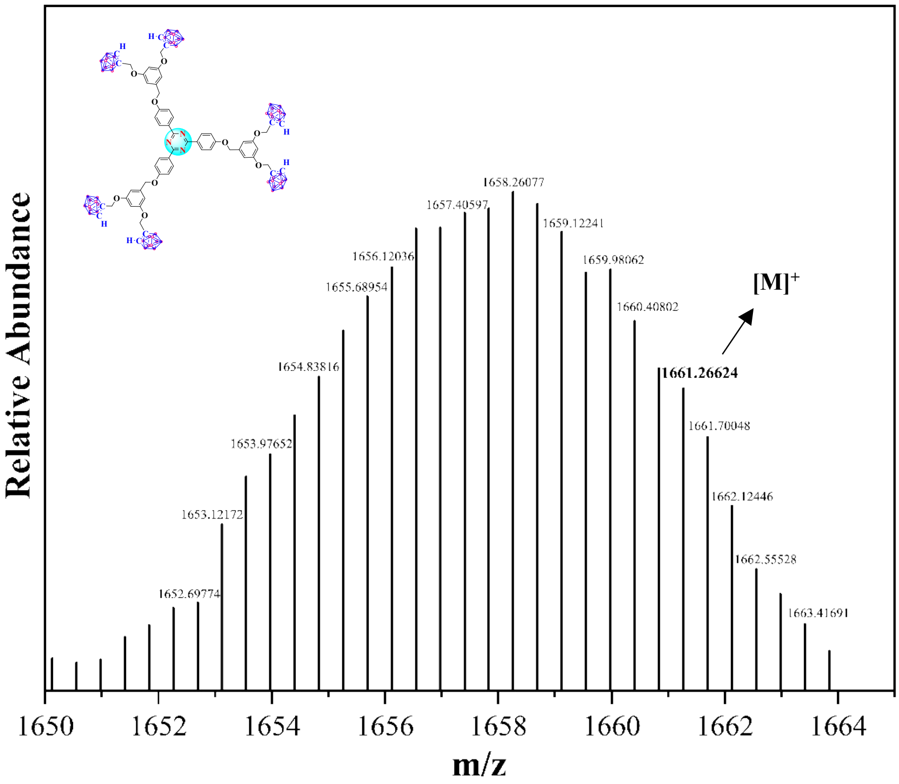

Figure S21: Mass spectra of compound **Tz-6-CB.**

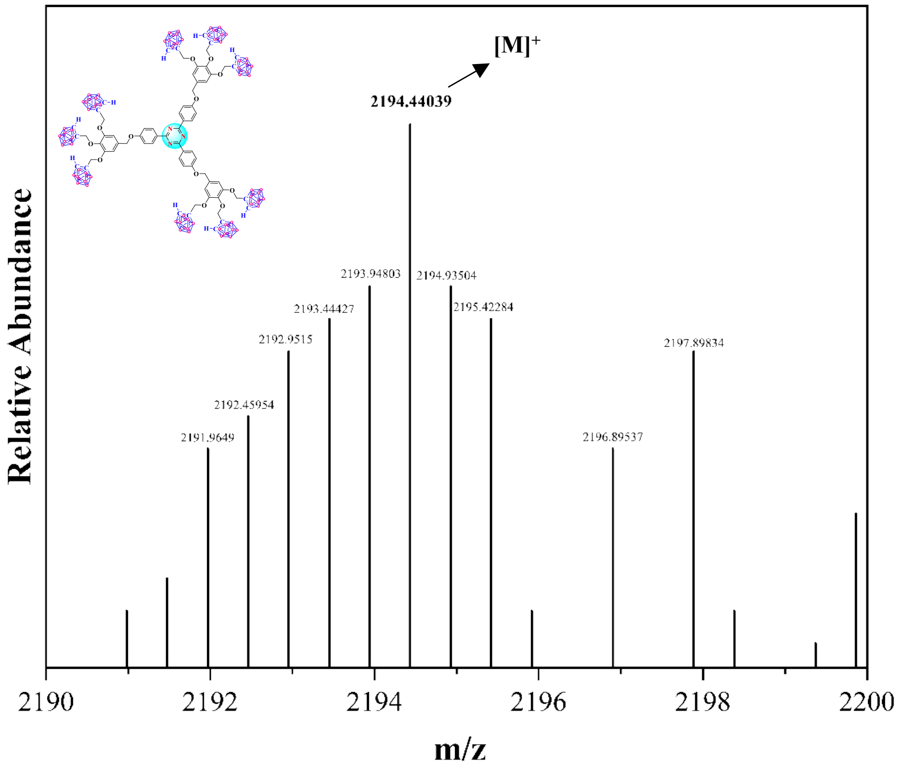

Figure S22: Mass spectra of compound **Tz-9-CB.**

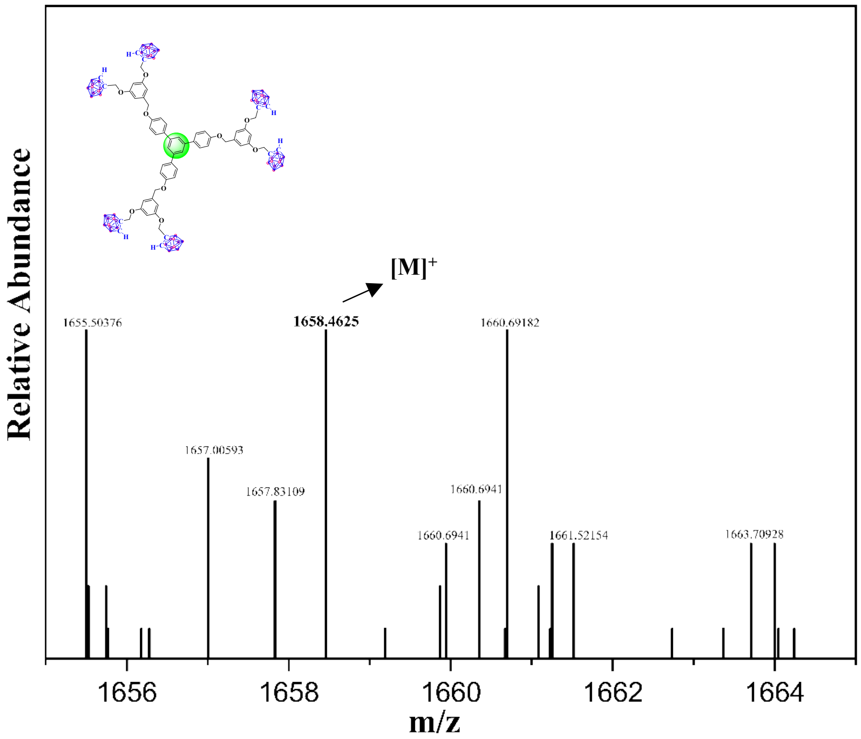

Figure S23: Mass spectra of compound **Ph-6-CB.**

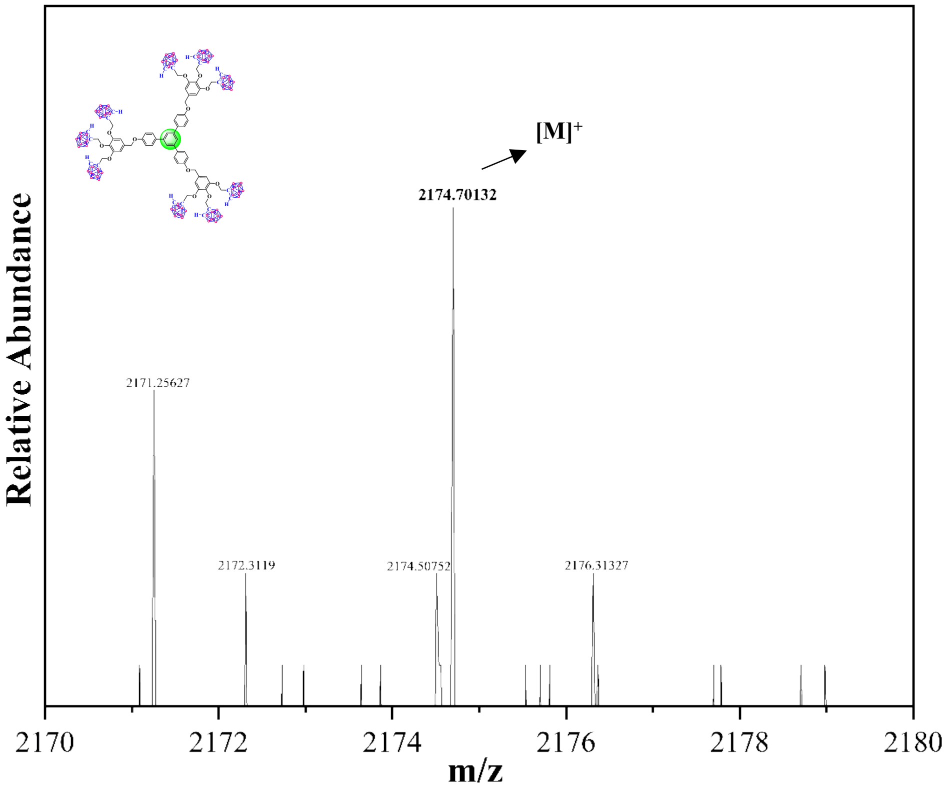

Figure S24: Mass spectra of compound **Ph-9-CB.**

Additional Computational Data

The following tables provide the complete set of conceptual DFT reactivity descriptors (**Table S1**) and the higher-lying singlet excited states (**Table S2**) for the four dendritic carborane-containing trimers **Ph-6-CB**, **Ph-9-CB**, **Tz-6-CB**, and **Tz-9-CB**, complementing the data presented in the main text (Tables 3 and 4, respectively). All values were obtained at the B3LYP/6-31G level of theory using the Gaussian 16 software package, with IEF-PCM (acetone, $\varepsilon = 20.493$) employed as the solvent for the TD-DFT calculations. As discussed in Section 2.3 of the main manuscript, these values are presented to illustrate qualitative trends across the series; absolute numerical values are model-dependent and are not intended for quantitative comparison with experimental observables.

Molecular Properties	Mathematical Description	Energy (eV)			
		Ph-6-CB	Ph-9-CB	Tz-6-CB	Tz-9-CB
Energy of LUMO	E_{LUMO}	-1.0231	-1.3401	-2.0373	-2.4033
Energy of HOMO	E_{HOMO}	-5.8888	-6.1954	-6.3710	-6.7865
Energy Gap	$\Delta E_g = E_{LUMO}-E_{HOMO}$	4.8657	4.8553	4.3327	4.3832
Ionization Potential (I)	$I = - E_{HOMO}$	5.8888	6.1954	6.3710	6.7865
Electron Affinity (A)	$A = - E_{LUMO}$	1.0231	1.3401	2.0373	2.4033
Electronegativity (χ)	$\chi = - (E_{LUMO} + E_{HOMO})/2$	3.4559	3.7677	4.2041	4.5949
Chemical potential (μ)	$\mu = (E_{LUMO} + E_{HOMO})/2$	-3.4559	-3.7677	-4.2041	-4.5949
Global Hardness (η)	$\eta = (E_{LUMO} - E_{HOMO})/2$	2.4328	2.4276	2.1663	2.1916
Global Softness (S)	$S = 1/2\eta$	0.2055	0.2059	0.2308	0.2281
Global Electrophilicity index (ω)	$\omega = \mu^2/2\eta$	2.4543	2.9228	4.4725	4.8158
Global Nucleophilicity index (N)	$N = 1/\omega$	0.4074	0.3421	0.2235	0.2076

Table S1: Complete set of conceptual DFT reactivity descriptors derived from the computed HOMO and LUMO energies of **Ph-6-CB**, **Ph-9-CB**, **Tz-6-CB**, and **Tz-9-CB** at the B3LYP/6-31G level.

Molecules	S_n	Energy (eV)	λ (nm)	f	Dominant Transition
**Ph-6-CB**	S_3	4.5030	275.34	0.8982	H → L, H → L+1
	S_4	4.5901	270.11	0.0010	H → L
	S_5	4.7374	261.72	0.2768	H–1 → L
**Ph-9-CB**	S_3	4.5153	274.59	1.0776	H → L, H → L+1
	S_4	4.6030	269.36	0.0540	H → L

	S_5	4.6833	264.73	0.0000	H–1 → L+1, H–1 → L+2
**Tz-6-CB**	S_3	3.8390	322.96	0.7506	H → L, H → L+1
	S_4	3.8662	320.69	0.9900	H → L
	S_5	3.8826	319.33	0.0663	H → L+1, H–1 → L
**Tz-9-CB**	S_3	3.8566	321.49	1.2503	H–1 → L, H–1 → L+1
	S_4	3.8698	320.39	0.4552	H → L, H → L+1
	S_5	3.8749	319.97	0.0803	H–2 → L+1

Table S2: Higher singlet excited states (S_3–S_5) of **Ph-6-CB**, **Ph-9-CB**, **Tz-6-CB**, and **Tz-9-CB** obtained from TD-DFT calculations at the B3LYP/6-31G level with IEF-PCM (acetone, $\varepsilon = 20.493$).
